# Employing fluorine for supramolecular control in self-assembled and self-organised molecular systems

**DOI:** 10.1039/d5sc05945c

**Published:** 2025-11-04

**Authors:** Duncan W. Bruce

**Affiliations:** a Department of Chemistry, University of York Heslington York YO10 5DD UK duncan.bruce@york.ac.uk +44 (0)1904 324085

## Abstract

This perspective will consider some of the ways in which the judicious deployment of individual fluorine atoms or collections thereof in molecular systems can influence their supramolecular organisation. While many of the examples will be from liquid crystal chemistry, the aim is to illustrate the effects of fluorine substitution in such a way to inspire a more general deployment strategy.

## Abstract 2

As a substituent in a molecular material or as part of a perfluoroalkyl chain, judicious use of fluorine can have a profound effect on the properties of molecular materials, in particular liquid crystals.

## Graphical abstract

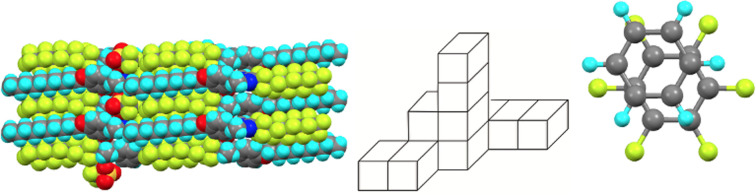

## 1. Introduction

Fluorine is a remarkable element. Its small atomic size and large nuclear charge confer the largest first ionisation energy of any element excluding the rare gases He and Ne, as well as the largest electronegativity of any element and the second largest electron gain enthalpy. As such, its introduction into molecular design can have a rather large effect for sometimes even small levels of incorporation. This has been exploited in numerous areas of chemistry that are not the focus of this perspective, but which include, for example, biomedicine,^1,2^ biomolecular science,^3,4^ synthetic^5–8^ and catalytic chemistry.^9–15^ However, the focus of this article is the way in which fluorine may be deployed in molecular materials chemistry as exemplified in part by its deployment in the context of thermotropic liquid crystals.

**Fig. 1:**
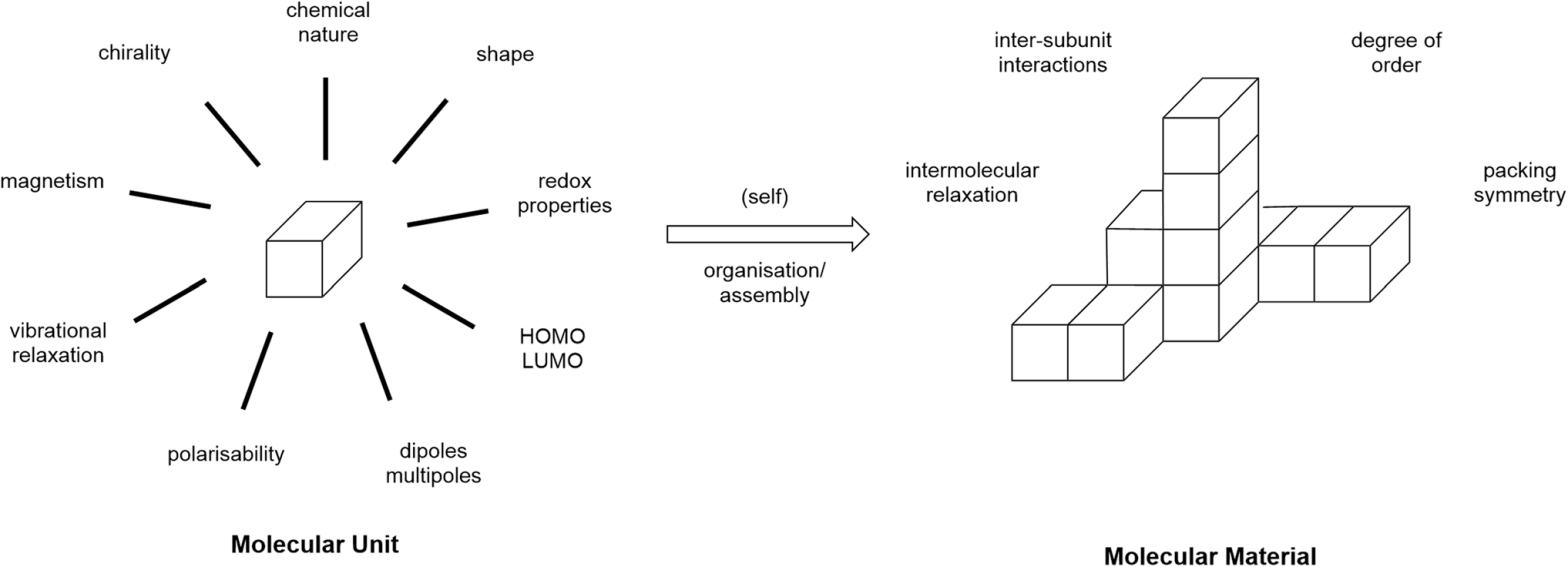
Adaptation of a figure from ref. 16 to illustrate Simon's definition of a molecular material.

In the mid-1980s, Simon co-authored a series of three overview articles^16–18^ in which he sought to consider how molecular materials may be deployed and, in the first of these, he offered the following definition: ‘Molecular materials are constituted of molecular units which can be isolately synthesized and which are, in a second step, organized into some condensed phase.’ This definition was accompanied by a diagram, an adapted version of which is reproduced as Fig. 1.

This model is a valuable and insightful way to think about the expression of molecular properties in a bulk material and recognises the fundamental importance of the organisation of the molecular sub-units into that bulk material. In addition, of course, while the physical properties themselves are then influenced at the molecular level and then subsequently through organisation, molecular-level changes will have an effect on that organisation and so some of the effects can end up being rather subtle.

Consider the following example. The dipole moment induced (*µ*_i_) in a molecular species on interaction with an external field (*E*) is given by:*µ*_i_ = *αE* + *βE*^2^ + *γE*^3^ + …where *α* is the linear molecular polarisability and *β* and *γ* are the first and second molecular hyperpolarisabilities, respectively, and where *α* ⋙ *β* ⋙ *γ*. Molecules with large values of *β* can double the frequency of light with which they interact and it is found that non-zero values of *β* are obtained only for molecules that are non-centrosymmetric (4-aminonitrobenzene is an archetypal example). Well-developed design rules have emerged to optimise *β* in molecular species.

Of course, in order to use frequency doubling practically, then account must be taken of the macroscopic response – *i.e.* in the condensed phase – which is expressed in terms of the macroscopic induced polarisation, *P*_i_ such that:*P*_i_ = *χ*^(1)^*E* + *χ*^(2)^*E*^2^ + *χ*^(3)^*E*^3^

It is then also the case that to obtain non-zero values of *χ*^(2)^, the non-centrosymmetric molecules must also crystallise in a non-centrosymmetric space group (note that *β* and *χ*^(2)^ are related).

Referring back to the definition proposed by Simon *et al.*, molecules can, therefore, be ‘isolately synthesized’ using the known design rules to maximise the molecular hyperpolarizability, *β*. However, the second step in which they are ‘organized into some condensed phase’ must result in a non-centric crystal structure for if it does not, then however high the molecular response that has been engineered, the macroscopic effect will be zero. The challenge is then to optimise *β* while including molecular design features that affect intermolecular interactions, promote a non-centric solid-state arrangement and so allow for non-zero values of *χ*^(2)^. Both of these are achieved by molecular design and the subject is eloquently described in more detail in ref. 19.

In this perspective, it is intended to show how incorporation of fluorine in different guises impinges on the properties of (mainly) liquid-crystalline (LC) materials along with some ionic liquids (ILs). This is a good way in which to illustrate the power of Simon's approach for all the properties of interest are associated with the bulk phase, most particularly the way in which individual molecules self-organise and how that it expressed in different physico-chemical properties. The aim is that the descriptions do not require a detailed knowledge of liquid crystals, even if occasionally some of the concepts are a little more involved than others. To that end, the use of some of the more common specialist terms will be minimised in favour of a slightly more descriptive approach. Nonetheless, there will be aspects that can be better appreciated with some elementary knowledge and so some introductory material is found in the SI, which shows structural representations of the main LC phases discussed.

## 2. Exploiting fluorine's electronegativity

### 2.1 Quadrupoles

**Fig. 2:**
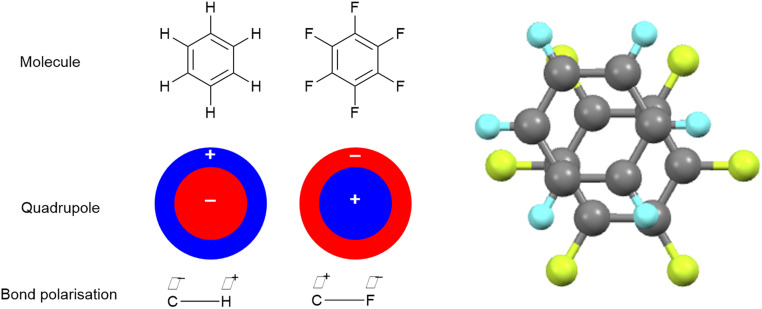
Co-crystal formation between benzene and hexafluorobenzene and the crystal structure of phase III of the molecular complex obtained at 225 K.^21^

While a very effective π-donor in the correct circumstances, as the most electronegative element fluorine is most commonly associated with its ability to attract electrons to itself inductively through σ-bonds. This can have different consequences. For example, compare benzene and hexafluorobenzene (Fig. 2). While the C–F bond is strongly polarised with electron density accumulating on F (*χ*(C) − *χ*(F) = −1.43), the C–H is polarised in the opposite direction and to a much lesser degree (*χ*(C) − *χ*(H) = 0.35). Consequently, the quadrupoles for the two molecules are complementary, which means that they can interact attractively. This phenomenon was reported by Patrick and Prosser^20^ in 1960 who reported a 1 : 1 molecular complex (Fig. 2), which melts at 23.7 °C while the individual components each melt at *ca.* 5 °C. Note that this is a quadrupolar interaction and is not charge-transfer in nature. While the results are too numerous to cite here, the Cambridge Crystallographic Database contains many more examples of very similar motifs.

While this result is of interest for structural reasons, there is also practical benefit to be exploited. Thus, it was determined previously that a low-yielding Williamson ether alkylation of pentabromophenol^22^ could be improved by the addition of a small amount of benzene as a co-solvent with DMF.^23^ The thought was that C_6_Br_5_OH would have a quadrupole of the same topology as that of C_6_F_6_, allowing the formation of an association complex capable of enhancing solubility. This small change to the conditions doubled the yield to around 75%.

**Fig. 3:**
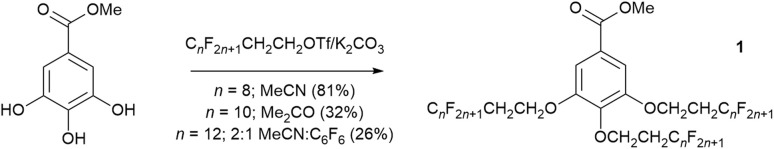
Conditions for the formation of methyl 3,4,5-tris(semiperfluoroalkyloxy)benzoates (1) as a function of the length of the perfluoroalkyl chain segment.

A similar approach was later adopted in the preparation of methyl benzoates that were 3,4,5-trisubstituted with semiperfluoroalkyl chains (OCH_2_CH_2_C_*n*_F_2*n*+1_). With increasing length and/or number of fluoroalkyl chains, their solubility can be a real issue, and various fluorous co-solvents such as trifluoromethylbenzene^25^ and perfluoro-1,3-dimethylcyclohexane have been deployed previously to aid solubility.^26^ Thus, while it was possible to prepare methyl 3,4,5-trisubstituted benzoates (1, Fig. 3) for *n* = 6 and 8 in acetonitrile or acetone, respectively, where *n* = 12 the reaction stopped at disubstitution owing to the insolubility of the compound. In order to get the reaction to proceed, therefore, it was necessary to use a 2 : 1 mixture of acetonitrile:hexafluorobenzene as solvent in order for the reaction to go in even a low yield of 26% (Fig. 3). Once more, the enhanced solubility of the disubstituted compound, which allows the reaction to proceed, is ascribed to the formation of a quadrupolar association complex.^24^

Another consequence of the difference in electronegativity when comparing C–H and C–F bonds, is that the smaller difference in the former case means that the electrons associated with the C–H bonds are polarisable, whereas in the latter case, the very big difference in effect means that the bonds are polar. Thus, the greater polarisability in hydrocarbons is seen in the refractive index, which is 1.37 for hexane^27^ compared with 1.25 for perfluorohexane despite the much greater number of electrons in the latter.^28^ It is also seen in the magnitude of dispersion forces as, despite its much greater molecular mass (338 g mol^−1^ for perfluorohexane *vs.* 86 g mol^−1^ for hexane), the boiling point of C_6_F_14_ is 57 °C compared with 69 °C for hexane.

### 2.2 Halogen bonding

If hexafluorobenzene is subject to the replacement of one fluorine by iodine, then the inductive electron-withdrawing effect creates an extremely electrophilic iodine. This has been exploited in the phenomenon known as halogen bonding.^29^ Thus, while there are examples of halogen bonding that go back to the 19th century,^30–32^ it was first delineated in the solid state by Hassel and co-workers^33,34^ who prepared and characterised crystallographically a series of complexes between bromine and 1,4-dioxane. The phenomenon was then studied extensively in the gas phase by Legon.^35,36^ However, its exploitation in supramolecular chemistry^37^ was led in Milan by Metrangolo, Resnati and many co-workers.^38^

**Fig. 4:**
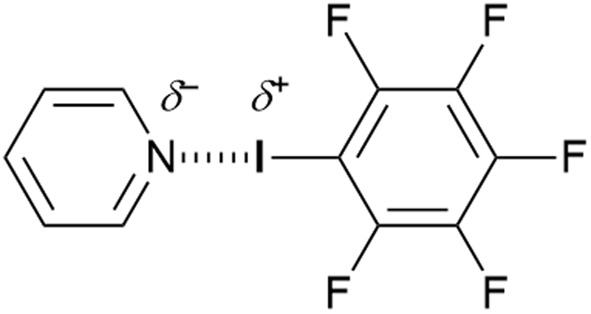
Classical halogen bonding motif featuring a Lewis base (pyridine) and an electrophilic halogen.

Thus, attaching the polarisable iodine to a strongly electron-withdrawing group such as C_6_F_5_ or a perfluoroalkane leads to the development of a positive electrostatic potential on iodine, which tends to be quite localised directly opposite to the C–I bond. This is shown in calculations reported by Clark *et al.* and led to the concept of the σ-hole,^39^ which can then interact attractively with a pair of electrons from a Lewis base such as pyridine to form a non-covalent complex (Fig. 4).

**Fig. 5:**
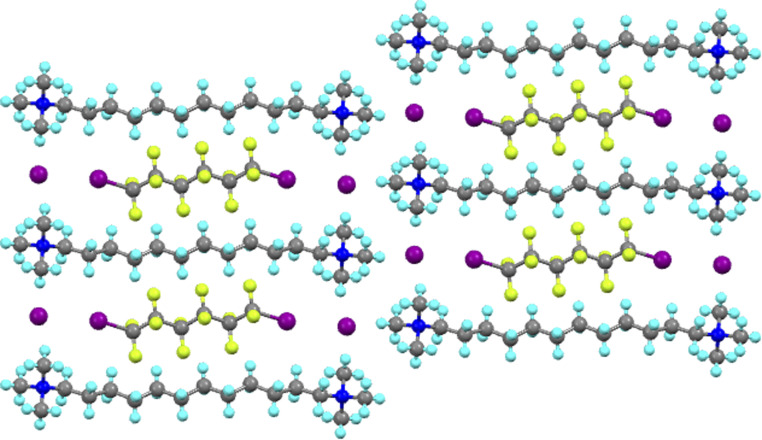
View down the *a*-axis in the structure of the co-crystal formed between I(CF_2_)_6_I and [Me_3_N–(CH_2_)_12_–NMe_3_]I_2_.

The Milan group led the exploitation of this phenomenon and it is not possible here to do justice either to their work or to the many other groups who have produced such beautiful and elegant chemistry using this interaction. However, one of many noteworthy examples is their demonstration of dynamic separation of individual components from mixtures of α,ω-diiodoperfluoroalkanes using bis(trimethylammonium)alkane diiodides.^40^ Thus and as shown in Fig. 5 for I(CF_2_)_6_I co-crystallised with the iodide salt of 1,12-bis(trimethylammonium)dodecane, the bis(trimethylammonium)alkane dications and their iodide anions are arranged so as to form a rectangular pocket with dimensions of ≈16.4 × 9.6 Å measured using the nitrogen atoms. Into this pocket fits a diiodoperfluoroalkane owing to the halogen bond to the iodide anions at each end, resulting in an iodide to iodide distance of ≈17.1 Å. In fact it was observed that I(CF_2_)_*n*_I would co-crystallise with the iodide salt of a dication of formula [Me_3_N–(CH_2_)_*n*+6_–NMe_3_]^+^ with very good selectivity.

Very significantly, it was also observed that allowing the vapour of the diiodoperfluoroalkane to come into contact with a solid sample of the appropriate bis(trimethylammonium)alkane iodide resulted in the formation of the same co-crystal as obtained from solution. This takes on added importance when it is realised that the starting ammonium salt does not have a porous structure. This represents an attractive approach to the separation of diiodoperfluoroalkanes of different chain lengths, which are obtained as a mixture in the manufacturing process.

**Fig. 6:**
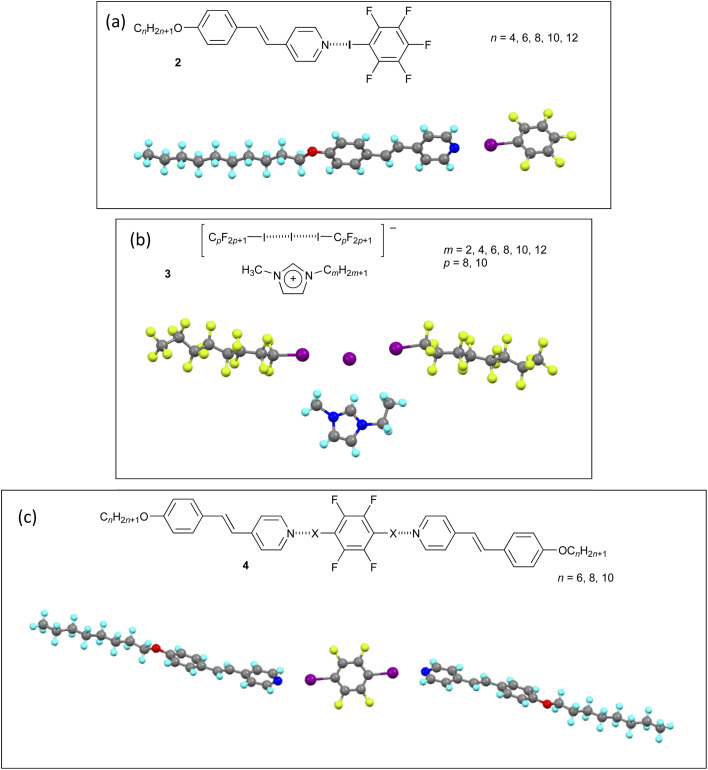
Three examples of liquid-crystalline materials formed using halogen bonding, each with a single crystal structure: (a) 4-alkoxystilbazole with iodopentafluorobenzene (2); (b) 1-methyl-3-alkylimidazolium salt of the halogen-bonded anion derived from two equivalents of a perfluoroalkyliodide and the iodide anion (3); (c) 2 : 1 complex between a 4-alkoxystilbazole and 1,4-diiodotetrafluorobenzene (4).

One way in which halogen bonding has been developed is in the design of liquid-crystalline systems, with the first examples prepared using 4-alkoxystilbazoles and iodopentafluorobenzene (2; Fig. 6).^41^ Neither component has LC properties, but the halogen-bonded complexes show a nematic phase for *n* = 4 and 6 and a SmA phase††Nomenclature and abbreviations relating to liquid crystal mesophases are found in the SI: N is used for nematic phase and SmA for smectic A, SmC for smectic C *etc.* for *n* = 6, 8, 10 and 12, persisting up to 84 °C for *n* = 12.

Many more examples followed and are collected in a recent review,^42^ but the following perhaps warrant a small, additional mention. The ionic materials (3) shown in Fig. 6 ^43^ are interesting as they are liquid crystals for small values of *m*, which means that the liquid crystallinity originates in the complex anion formed by halogen bonding of iodoperfluoroalkanes to an iodide anion (same interaction as described in the separation of diiodoperfluoroalkanes above). The necessary anisotropy here undoubtedly arises from the rigidity of the perfluorocarbon chains, representing a rare example of thermotropic liquid crystal behaviour driven by an anion.

The trimeric 2 : 1 complexes (4) shown in Fig. 6 were prepared and crystallised for X = I and Br and the two structures as determined by X-ray diffraction were very similar.^44^ However, whereas the complex with X = I showed monotropic‡‡The term monotropic means that a phase is found only on cooling below the melting point. As such, it is thermodynamically unstable. nematic phases (*n* = 6, 8 and 10), when X = Br (*n* = 8) the complex simply melted directly to the isotropic liquid and no mesophase was seen on cooling. Thus, while the N⋯Br halogen bonds were strong enough to allow the co-crystals to form, at the melting point of just above 90 °C, they evidently ruptured meaning that no LC phase was observed In this case, therefore, the lability of the halogen bond would seem to be determined not by the electron-withdrawing power of the fluorine atoms on the benzene ring, rather by the lower polarisability of bromine compared with iodine.

This prompts two additional comments. First is that in the vast majority of cases studied, the halogen bond is thermally labile once in a molten state and particularly in the isotropic liquid state. Direct evidence was found for this behaviour in 2 : 1 complexes formed between alkoxystilbazoles and 1,3-diiodotetrafluorobenzene.^42,45^ Second is that great care must be taken in preparing halogen-bonded LCs. Correct stoichiometry is crucial as otherwise the material is, by definition, impure as it will contain and excess of one component and so the physico-chemical properties are those of a mixture. The precise stoichiometry is best achieved by growing crystals. Where that is not possible, then mixing the carefully weighed components in the melt can offer a reasonable alternative,^46^ although liquid-assisted grinding cannot.^47^

**Fig. 7:**
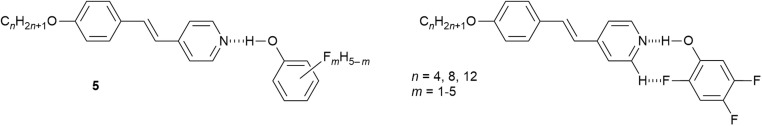
Left: Structure of the hydrogen-bonded fluorophenol complexes of alkoxystilbazoles; right: intramolecular hydrogen bonding to generate an additional ‘ring’ in the structure.

Finally here, it is noted in passing that replacing hydrogen by fluorine in fluorophenols increases the acidity of the phenol and makes them amenable to the formation of hydrogen-bonded complexes with alkoxystilbazoles, which were also liquid crystalline.^48^ Forty-eight complexes (5) were prepared and characterised based on *n* = 4, 8 and 12, and using 16 of the 19 isomers of the fluorophenols, with ten isomers with *n* = 8 characterised crystallographically. It was determined that the LC phases were most stable when there was the structural possibility to form an extra intramolecularly, hydrogen-bonded ‘ring’ system as shown in Fig. 7 for a 2,4,5-trifluorophenol complex. It was also determined that the p*K*_a_ of the phenol was not in any way related to the clearing point of the LC phase, demonstrating that the clearing transition to the isotropic liquid is not driven by rupture of the hydrogen bond.

## 3. Fluorine as a small, polar atom in rod-like liquid crystals

### 3.1 Tuning the mesomorphism and physical properties of small-molecule, device-related liquid crystal materials

In the study of rod-like liquid crystals, the incorporation of lateral substituents can have a huge effect. They act to reduce the molecular anisotropy, which can have the effect of changing the phase behaviour and/or significantly destabilising, even suppressing totally, any observed mesophases. However, the relatively small size of F (covalent radius = 0.57 Å) when compared to H (covalent radius = 0.31 Å),^49^ means that its use as a lateral substituent is least disruptive of any functional group and, more than that, its high electronegativity can have a profound effect on dielectric properties. This area has been the subject of its own reviews^50–53^ and, as it is very liquid crystalline in flavour, just two examples – fluorinated terphenyls and a family of related polyfluorinated compounds with interesting nematic behaviour – will be chosen to show what is possible.

**Fig. 8:**
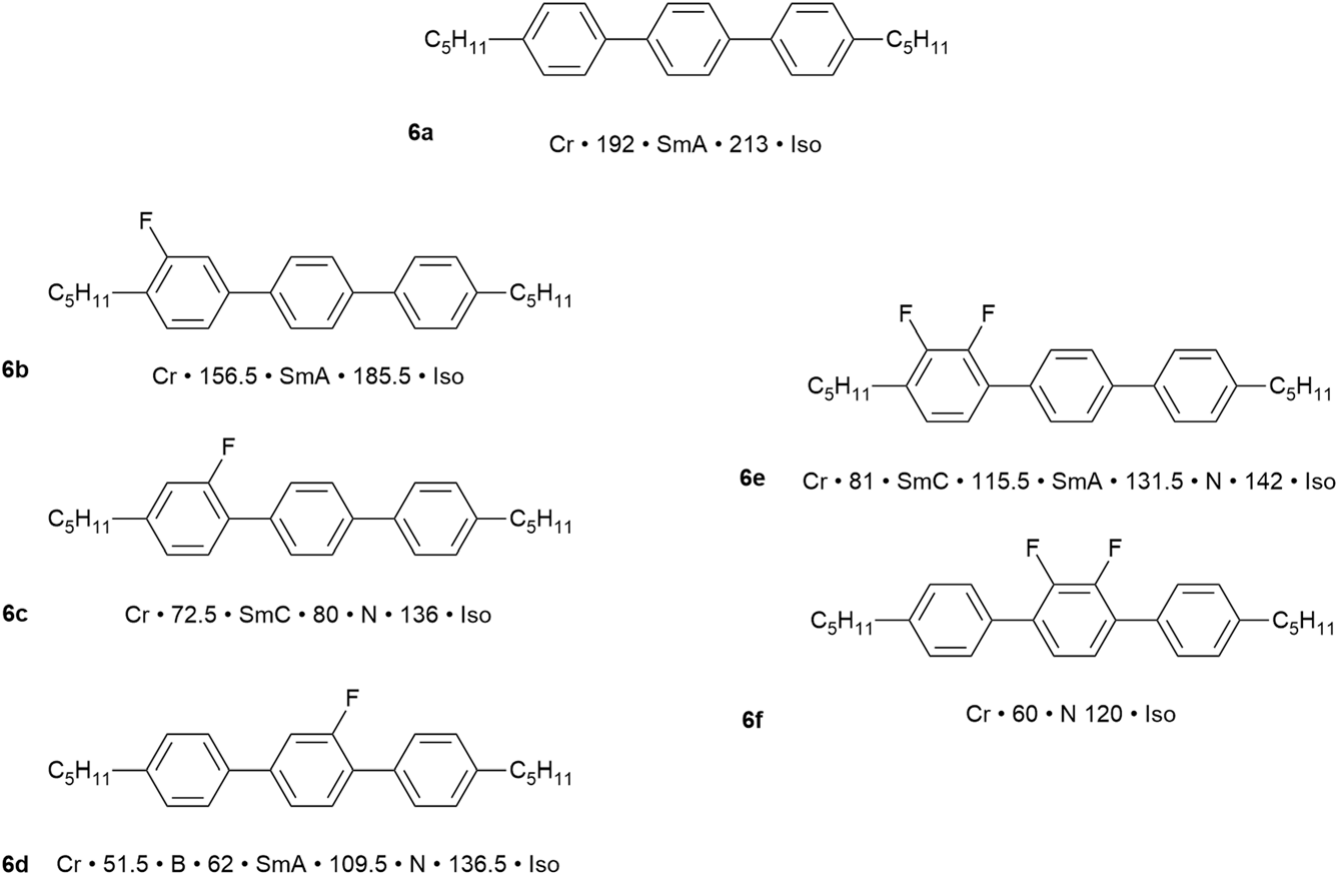
Examples of the effect on the mesomorphism of 4,4″-dipentyl-1,1′:4′,1″-terphenyl with different fluorosubstitution patterns.§§The thermal behaviour is read as (example for 4a): crystal (Cr) melts to give a SmA phase at 192 °C, when then clears to the isotropic liquid at 213 °C.

The synthesis of 4,4″-disubstituted terphenyls^54^ allows for the two terminal chains to be different, for them to be attached as alkyl or alkyloxy groups and for a range of regiospecific variations in the inclusion of fluorine substituents on the aromatic rings. This is, therefore, a vast structural and property space for exploration and so to show what is possible, just six examples are chosen as shown in Fig. 8.

The parent material, 4,4″-dipentyl-1,1′:4′,1″-terphenyl (6a), is a high-melting solid with a SmA phase that persists over 21 °C before clearing at 213 °C. In terms of monofluorinated derivatives, three regioisomers are possible and the difference in behaviour is significant. Thus, placing a single fluorine *ortho* to one of the terminal chains (6b) retains the same phases as the parent but with both the crystal and SmA phase destabilised by around 30 °C. However, move the fluorine one position round on the same ring so that it sits *meta* to the terminal chain (6c) and the effect is much more significant. Thus, the melting point comes down by 120 °C, the clearing point by 80 °C and the phase behaviour changes so that now a SmC and N phase are seen. Then, if the fluorine is moved onto the central ring (6d), the clearing point is unchanged with respect to (6c), but the nematic phase is stabilised, the SmA phase is re-introduced as the expense of SmC and a crystal smectic B phase is observed.

Being sterically close to the terminal chain, the F in 6b does not have a major effect on the mesomorphism and its position pointing away from the molecular core would tend to be favourable for smectic phase formation. However, when placed as a lateral substituent pointing inwards, the destabilisation is much greater, both due to a reduction in the anisotropy of the rigid molecular core and also because it introduces a lateral dipole that will also cause a degree of intermolecular repulsion. This will destabilise smectic phases (*ca.* 130 °C and 105 °C in 6c and 6d, respectively) and promote formation of the nematic phase (as observed).

Now consider just two materials, 6e and 6f, each containing an *ortho*-difluorinated phenylene ring. In 6e this is a terminal phenylene group and the mesomorphism shows a combination of the effects just described, so that transition temperatures are reduced, smectic behaviour is retained and a nematic phase is promoted. However, in 6f the difluorophenylene ring is now in the centre of the molecule, totally destabilising the smectic behaviour and leading to a nematic phase with a 60 °C range. There is also a significant reduction in the clearing point, which is now close to that observed for the other materials considered.

In the commercial application of liquid crystals in flat-panel displays, the range of property requirements that needs to be met is substantial and these are certainly not to be found in single components. For example, even the simplest of alphanumeric displays would be expected to function well while on holiday skiing (sub-zero temperatures) as well as in summers the like of which were experienced this year (2025) around much of Europe (up to 45 °C). Therefore, the range of desirable properties is engineered in by the use of mixtures. This subject of itself would also consume an entire review, but can be approached using two simple concepts. First, it is known that if a pure substance is ‘contaminated’ by the presence of another ‘impurity’, then its melting point will be depressed. Thus, mixing two or more compounds will lead to the depression of the melting point, extending the mesophase range down in temperature. Second is the observation in liquid crystals, that for two (or more) components with similar chemical nature, then both the clearing point and many physical properties (*e.g.* optical, magnetic, dielectric anisotropy) tend to be a reasonably linear function of composition.

**Fig. 9:**
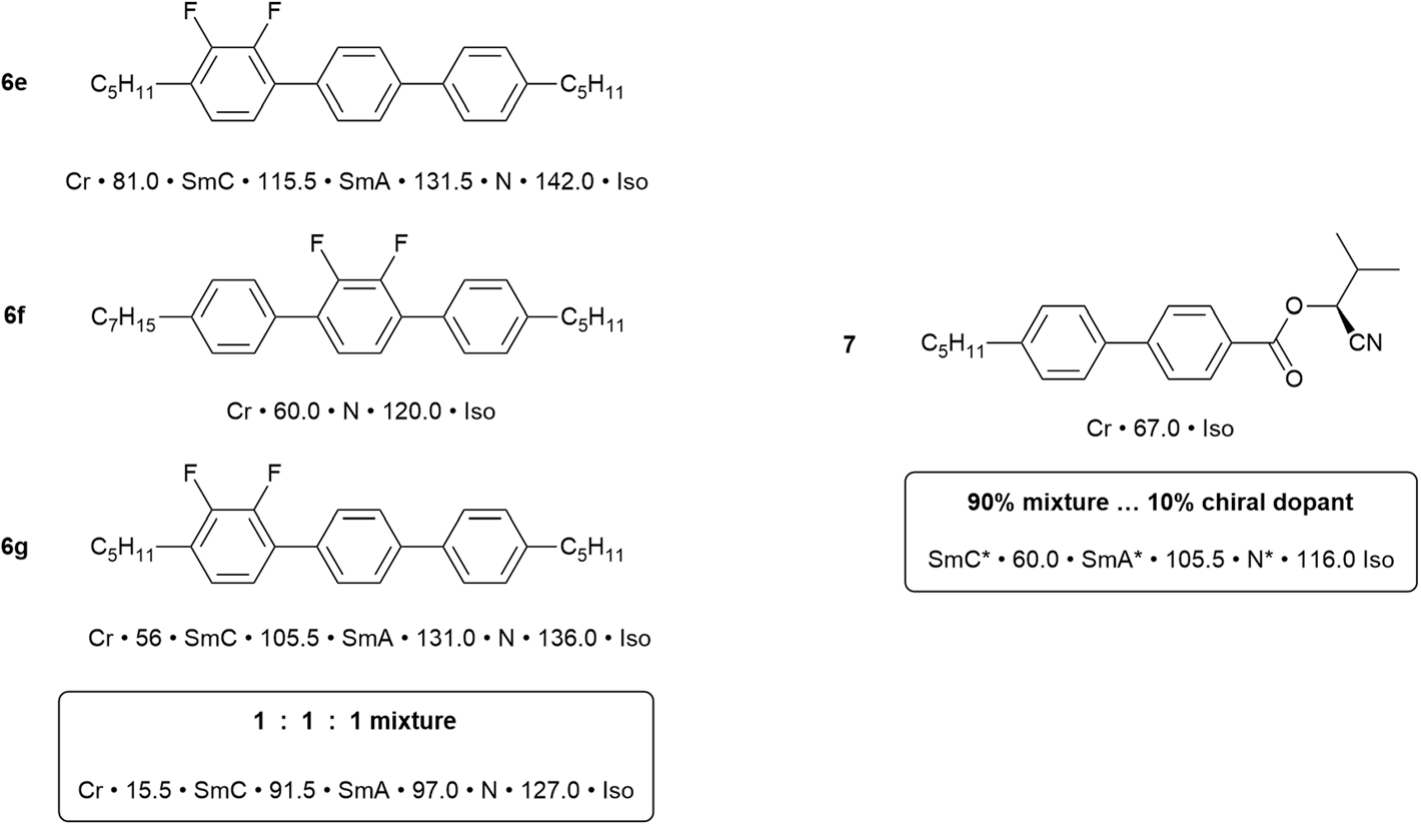
Left: Mixture components and mixture transition temperatures; right: chiral dopant and mixture temperatures.

Fig. 9 shows an example of a mixture prepare using two (6e and 6f) of the dipentylterphenyl compounds described above, plus a third (6g) in which the alkyl chains lengths are different. Having three components destabilises the crystal phase of the materials and the three phases of two of the components are retained. However, addition to the mixture of 10% of the chiral, non-LC compound (7, Fig. 9) has a dramatic effect, reducing the melting point well below ambient temperature and now producing a chiral SmC* phase (see SI) that extends up to 60 °C which is a reasonable upper limit for commercial application. Thus, the mixture has a negative dielectric anisotropy (see SI), which is required for application in devices based on the SmC* phase and a fast switching response time of 2 µs. Without going into detail of these physical responses, this example is indicative of what can be achieved and, at the time that this work was being done in the late 1980s/early 1990s, there was significant demand for ferroelectric SmC* materials for use in certain types of small display. The work and the lessons learned from it led to the formation of a spin-out company from the University of Hull – Kingston Chemicals – which operated successfully for nearly twenty-five years.

### 3.2 The ferroelectric nematic phase

**Fig. 10:**
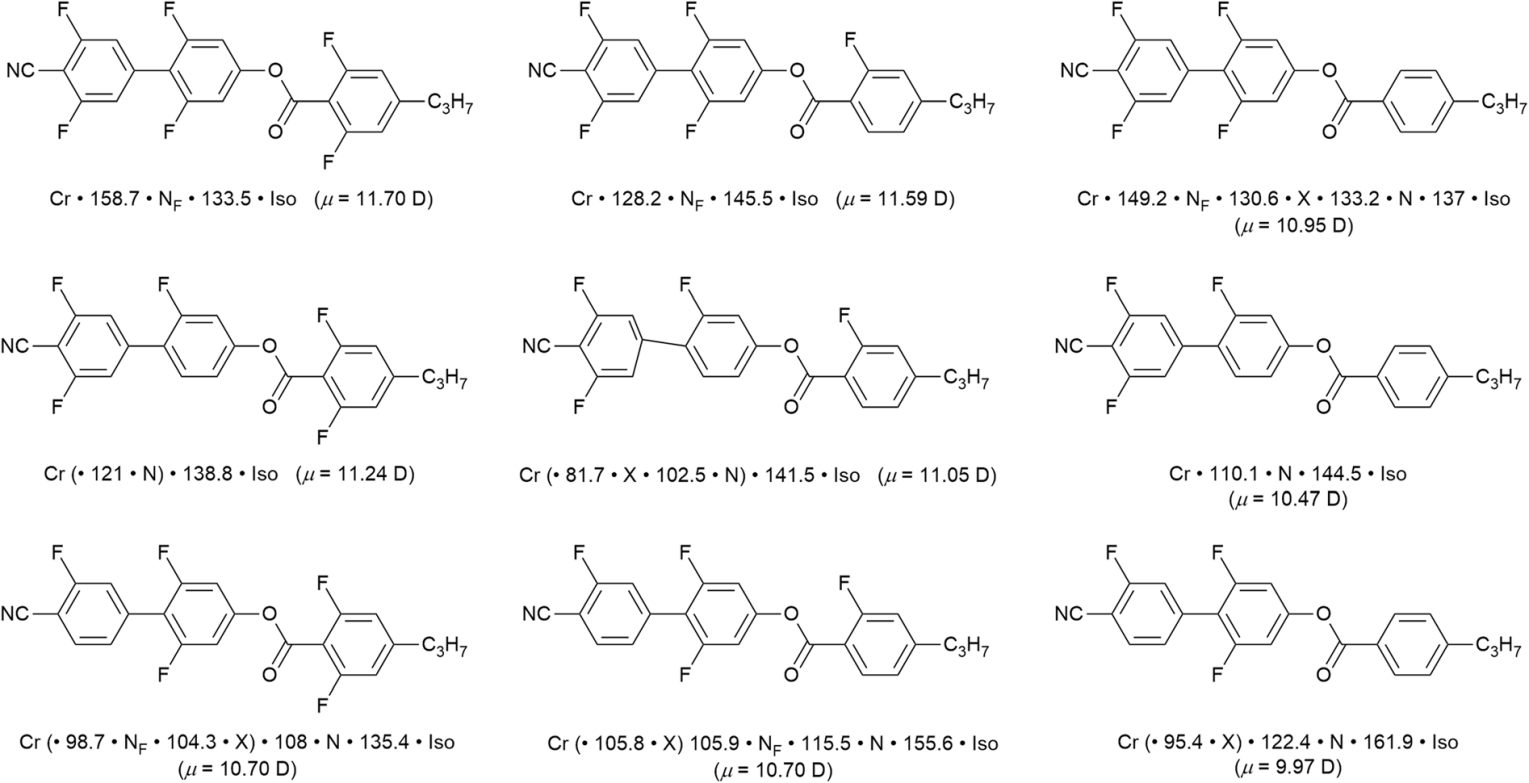
Series of nine related fluorinated cyanobiphenyl esters, their transition temperatures and calculated longitudinal dipole moment (Gaussian G16, B3LYP, GD3BJ/cc-pVTZ). The phase X exhibits an antiferroelectric response and is likely smectic in nature,^60^ but remains an active subject of investigation. Events recorded in brackets indicate monotropic behaviour.

To bring fluorine in small-molecule LCs a little more up to date, there has been something of a revolution in recent years by the identification of a ferroelectric nematic phase (N_F_).^55,56^ The materials that exhibit this phase are small, rod-like LCs that have a very large dipole moment along the long molecular axis, which in most cases arises from the presence of multiple fluorine substituents. As its name suggests, the organisation in the N_F_ phase requires total polar alignment of all of the dipoles and, while it is of great interest from a number of points of view,^57^ it is sufficient here to exemplify recently reported examples^58,59^ that show how different phases are tuned in and out as function of systematically modifying the pattern of fluorine substitution on the three-ring cyanobiphenyl propylbenzoate mesogen. The examples in Fig. 10, taken from ref. 58, indicate that the magnitude of the longitudinal dipole moment, which also serves as a proxy for the degree of fluorination, is not the major driving force in stabilising the N_F_ phase.

## 4. The use of perfluorocarbon chains – hydrocarbon/fluorocarbon separation

**Fig. 11:**
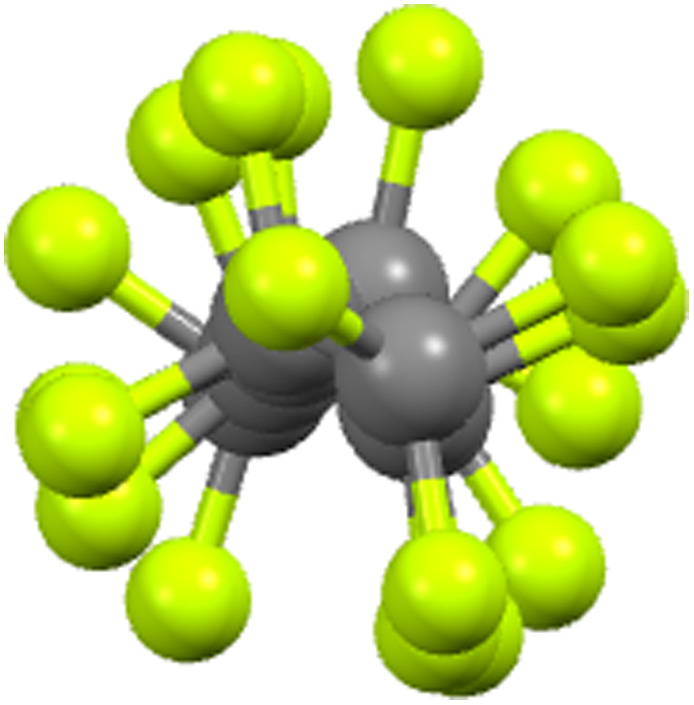
End-on view of the perfluorocarbon chain of 9-8F (see below) showing the helicity (re-drawn from the cif file).

Unlike hydrocarbon chains, which are flexible, perfluorocarbon chains are rather rigid on account of the greater size of F compared to H, this size difference sterically precluding flexibility. Indeed, more than that, in order to accommodate the steric demands of two fluorine atoms per carbon, a perfluorocarbon chain adopts a helical arrangement (Fig. 11) and as such, the chains are formally chiral. Both helical senses are found and so the materials are racemic.

Then there is a widely accepted paradigm that hydrocarbons are immiscible with fluorocarbons. Is this always the case? Somehow we learn that there is something known as a fluorous effect describing the observation that fluorocarbon chains tend to phase separate from hydrocarbon chains when the chains contain eight carbon atoms or more. However, it is difficult to find a place in the literature where the origin of this is both stated and explained. We had reason to be interested in this recently through a study of mixtures of ionic liquids, which has helped in our understanding of this phenomenon.

### 4.1 Ionic liquids and related compounds

**Fig. 12:**
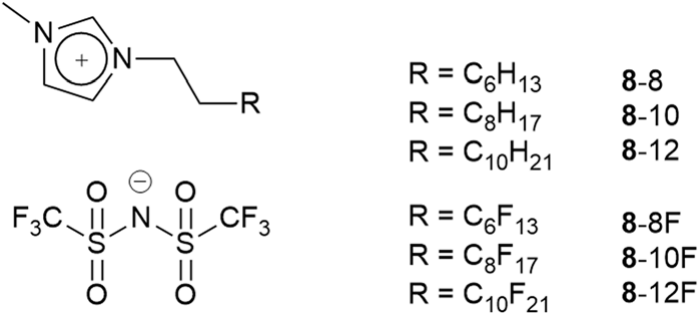
Some of the ionic liquid components of mixtures studied by surface and bulk techniques.

The synthetic possibilities offered in the preparation of ILs is huge, given the range of anions available and the synthetic flexibility associated with cations in particular, so that in principle, a particular set of physico-chemical properties might readily be ‘dialled in’ through molecular design. However, to make a bespoke salt each time is perhaps not the best use of time and resource and, therefore, a slightly more efficient strategy may be considered whereby IL mixtures are prepared and properties tuned in that way.^61^ Through a collaboration with groups using reactive atom scattering, we have undertaken a study of such mixtures^62–66^ and, most recently, we have considered mixtures where one component bears a hydrocarbon chain and the other a semi-perfluorocarbon chain (Fig. 12).

One of the driving forces for this work in IL mixtures is in the area of supported ionic liquid phase (SILP) catalysis.^67^ While Leitner and co-workers have pioneered the use of tethered ILs as supports to nanoparticulate metal catalysts,^68^ there is an alternative approach where a homogeneous catalyst is dissolved within an IL, which in turn coats a high-surface-area porous support such as silica. These latter systems are applicable in gas-phase catalysis and have reached pilot plant in their development in hydroformylation.^69^ Here, the use of IL mixtures offers control over factors such as bulk organisation and structure, as well as the nature of the gas–liquid interface. Both are important in the catalysis, for example in relation to the solvation of the catalyst itself and the transport of gases across the gas–liquid interface.

**Fig. 13:**
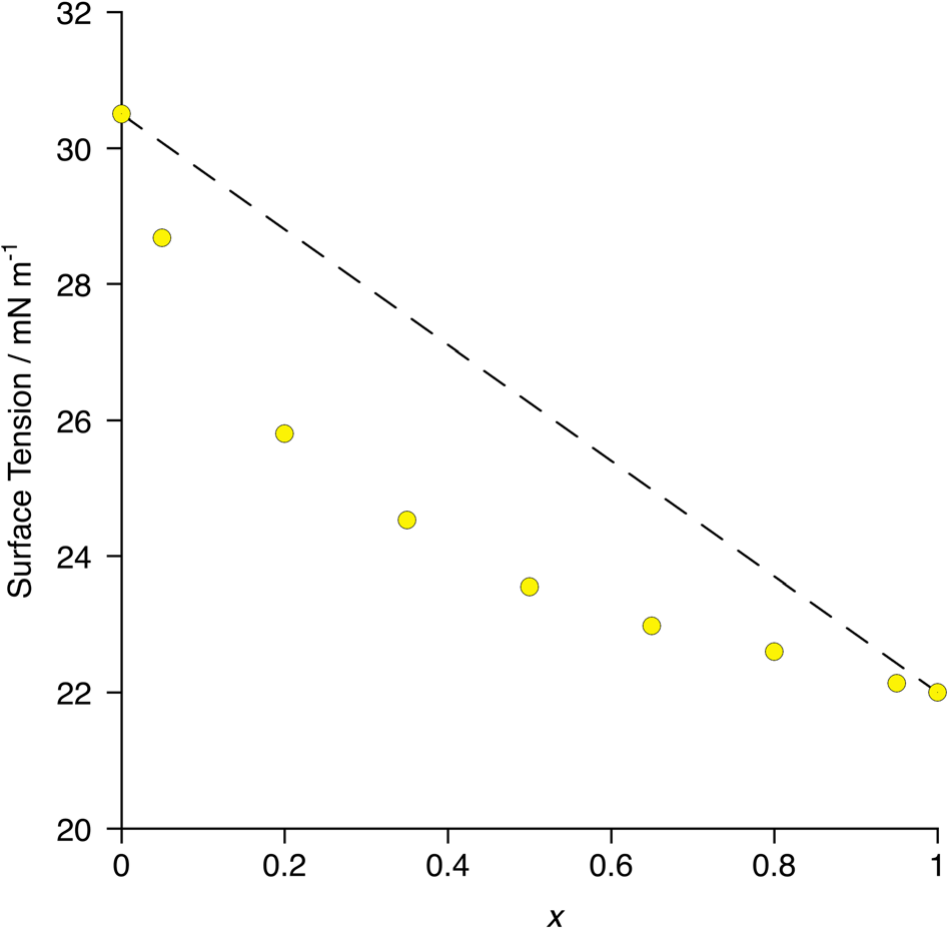
Plot of surface tension as a function of composition in the mixtures [8-8]_1−*x*_[8-8F]_*x*_. The dotted line represents a simple linear relationship with composition. Error bars are smaller than the plot symbols. Re-plotted from the published data in ref. 70.

In terms of surface behaviour, therefore, it was found that 8-8F enriches preferentially the surface of a mixture of 8-8 and 8-8F as exemplified by the more rapid change in surface tension with composition when compared with simple linear evolution (Fig. 13) – *i.e.* the deviation from linearity in the plot.^70^ The surface can, therefore, readily be made more fluorous with a relatively small amount of material, so influencing interfacial gas transport.

Then to begin to understand the behaviour of the mixtures in the bulk, two series of mixtures were prepared, namely [8-8]_1−*x*_[8-8F]_*x*_^71^ and [8-10]_1−*x*_[8-10F]^72^ (Fig. 12). In each mixture, the extended chain contained the same number of carbon atoms; this chain was hydrocarbon in one component and semiperfluorocarbon in the other. In both series at room temperature, the components were continuously miscible across the entire compositional range. However, note that while in the former series, the fluorocarbon chain segment is C_6_F_13_, in the latter it is C_8_F_17_, which may be expected to lead to phase separation.

**Fig. 14:**
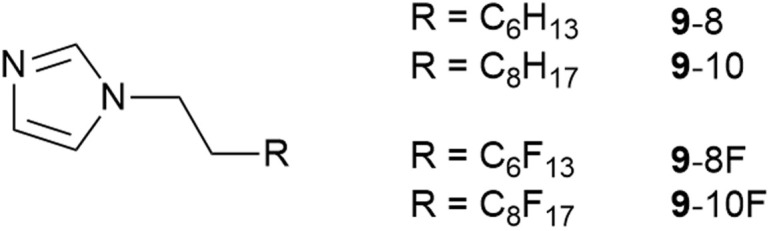
Neutral *N*-functionalised imidazoles related to the ionic liquids studied.

In order to understand this miscibility better, the synthesis of related neutral imidazoles (9, Fig. 14) was undertaken and mixtures were investigated using a combination of surface tension measurements, small-angle X-ray (SAXS) and neutron (SANS) scattering, complemented by atomistic molecular dynamics (MD) simulations.^73^

**Fig. 15:**
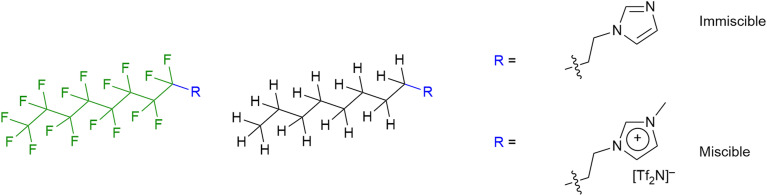
Visualisation of the imidazoles and methylimidazolium cations as simple substituted (perfluoro)alkanes.

The results showed that while mixtures of 9-8 and 9-8F were miscible across the compositional range, 9-10 and 9-10F were immiscible. Then compared with the results for the related ionic liquids, it seems that electrostatic attraction in the ILs is able to overcome the tendency of the pairs of materials to de-mix. Thus, considered in a simplified way, if the two components are considered as a substituted octane and a substituted perfluorooctane (Fig. 15), then for R = –CH_2_CH_2_–Im (Im = imidazole) the two components are immiscible, while for R = –CH_2_CH_2_–MIM^+^[Tf_2_N]^−^ (MIM^+^ = *N*-methylimidazolium) they are miscible.

**Fig. 16:**
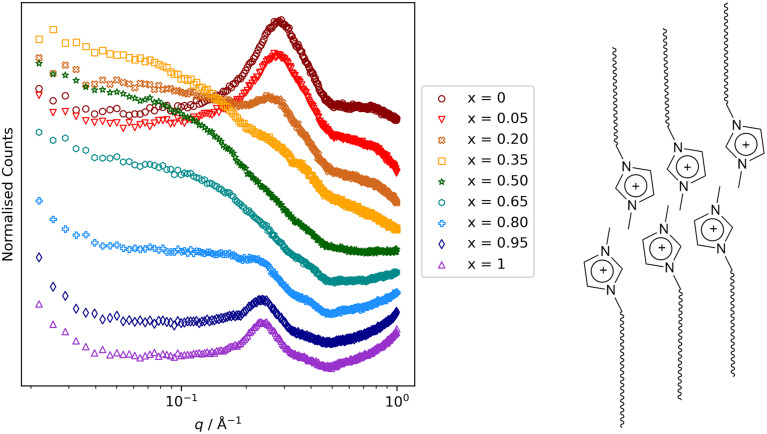
Small-angle neutron scattering data for the mixtures [8-10]_1−*x*_[8-10F]_*x*_. The reflection at *q* ≈ 0.3 Å^−1^ (≈21 Å) arises from very short-range correlations of local bilayer organisation of the long-chain ion pairs as illustrated schematically alongside (anions omitted for clarity). For intermediate values of *x*, it is therefore masked by scattering that persists to much smaller values of *q*, arising from the presence of small aggregates. Scattering figure reproduced from ref. 72.

Other key findings from this study that relate to hydrocarbon/fluorocarbon miscibility are as follows. First was that while small-angle scattering data for the mixtures [8-8]_1−*x*_[8-8F]_*x*_ were entirely straightforward, for [8-10]_1−*x*_[8-10F]_*x*_[Tf_2_N] and [9-8]_1−*x*_[9-8F]_*x*,_ the low-*q* region showed evidence of the formation of small aggregates (Fig. 16) for small and large values of *x* (*i.e.* small amounts of 8-8 in 8-8F or of 9-8 in 9-8F and *vice versa*). These observations were mirrored in the MD simulations and so while both mixture series showed total miscibility at all compositions, it is evident that some local segregation exists, which can be seen as foretelling the next observation. Thus, on lengthening the chains further to give 8-12 and 8-12F and 9-10 and 9-10F, neither pair showed miscibility of its components at room temperature. Thus, as described above, the immiscibility of 9-10 and 9-10F can be readily understood, whereas the immiscibility of 8-12 and 8-12F shows that when attempting to mix a perfluorodecane with a hydrocarbon equivalent, even the electrostatic attractions present in the IL are insufficient to overcome the unfavourable mixing that would otherwise result.

In this context, it is possible then perhaps to understand how the notion of a fluorous effect arises, for in a classic paper in 1967 containing the results of many careful measurements, Gilmour *et al.* evaluated the critical temperatures of mixing for a range of hydrocarbon/fluorocarbon mixtures.^74^ In the paper, they reported the critical temperature for hexane/perfluorohexane mixtures to be 22.8 °C, while for octane/perfluorooctane it is 75.4 °C. Thus, while the former pair mix at about room temperature, the latter do not and require heating (to what is in effect an upper consolute temperature) above 75.4 °C before they will do so. Therefore, for many individual materials or pairs of materials where there is a perfluorooctyl fragment, it would seem that the fluorous chains do prefer to self-associate.

Effective insight into the origin of this effect came from a 2019 computational study by Pollice and Chen.^75^ In what is a very detailed but well-executed paper, they argue (and this is very much a précis of a great deal of careful and detailed explanation) that (im)miscibility is the balance between the energetics associated with intermolecular H⋯H and F⋯F interactions balanced against two H⋯F interactions. The flexibility of hydrocarbon chains and the rigidity of fluorocarbon chains increasingly militates against intermolecular H⋯F interactions so that by the time a carbon chain is eight carbons long, it is energetically preferable to have two like interactions compared to two unalike interactions and the components are immiscible.

### 4.2 Influence on self-organisation in liquid crystals

**Fig. 17:**
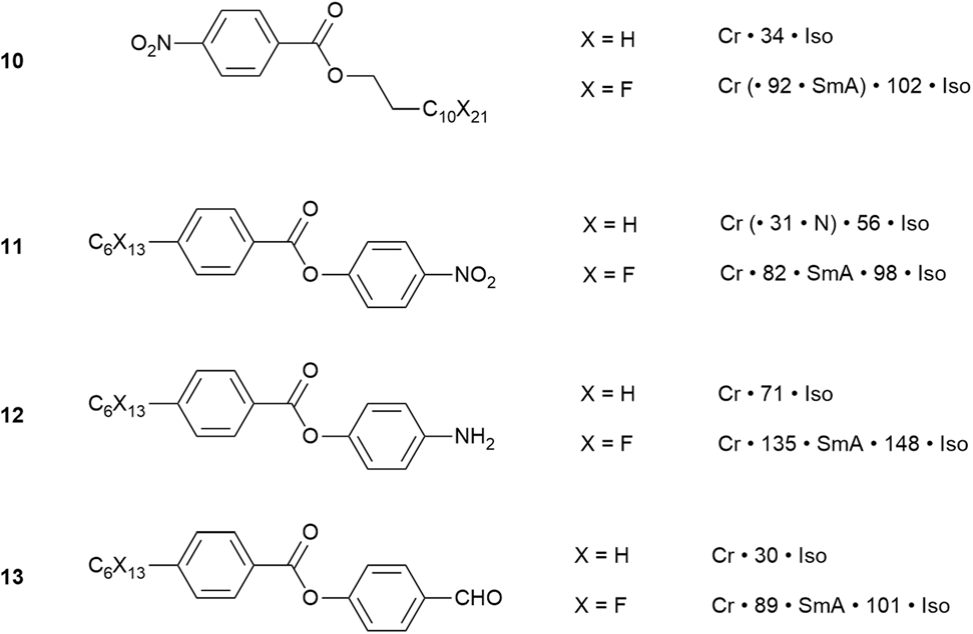
Comparison of the thermal behaviour for four pairs of compounds with hydrocarbon or fluorocarbon terminal chains and showing induction/enhancement of LC behaviour in the latter case.

In the generalised structure for rod-like liquid crystals presented in the SI, it is noted that one or both terminal groups (A and B in Fig. S2) is normally an alkyl chain, which could, of course, be replaced by a perfluorocarbon equivalent. It is found that such a substitution will certainly increase transition temperatures and can, on occasion, induce mesomorphism. This is illustrated by four pairs of compounds in Fig. 17.

For example, the simple alkyl ester of nitrobenzoic acid (10, X = H) melts to an isotropic liquid just above room temperature,^76^ whereas the use of a semiperfluoroalkyl chain (10, X = F) results in a very significant increase in the melting point (from 34 to 102 °C) and the induction of a SmA mesophase.^77^ On replacement of hydrocarbon by fluorocarbon chains, the other three pairs (11–13) show increases in transition temperatures in all cases, induction of LC behaviour in two (12, 13) and replacement of the N by a SmA phase in the other (11).^78^ Two observation come from these comparisons. First is that melting point and LC phase stability¶¶Note the LC phase stability relates to the upper temperature at which the phase exists and is unrelated to the range over which the phase exists. For example, a nematic phase existing over the range 30–60 °C is considered less stable than one existing between 85 and 90 °C. increase when the hydrocarbon chain is replaced by its fluorocarbon equivalent. This is related primarily to the reduction in chain mobility so that while hydrocarbon chains are rather mobile, which can act to suppress both mesophase and crystal phase formation, fluorocarbon chains are much more rigid and inflexible and so can have the opposite effect. Second is that in the nitro compound, the existing nematic phase is replaced by a SmA phase. This represents a rather general observation that is related in part to the possibility of self-association of fluorocarbon chains and also to the greater ease of organisation into a lamellar structure with a less flexible chain, which tends to favour smectic over nematic organisation.

### 4.3 Ionic liquids meet liquid crystals – evidence for triphilic organisation

**Fig. 18:**
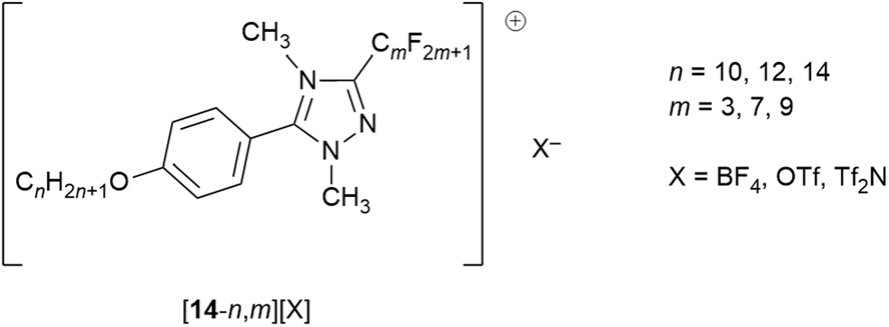
Structure of triazolium mesogens [14-*n*,*m*][X].

Having considered the behaviour of ionic liquids with fluorous chains and having introduced some basic ideas of how fluorous chains affect LC properties, what if the two are brought together? This can be done by considering a series of triazolium LCs – [14-*n*,*m*][X] – whose structure is shown in Fig. 18.^79^

In considering these cations, it is possible to identify three separate features that will influence the self-organisation into a LC phase. First is the hydrocarbon chains, then the fluorocarbon chains and finally the electrostatic charge. On the basis of the previous discussion, it might be expected that like chains self associate, while the charged regions will form another ‘domain’, leading to triphilic organisation. So it is that in common with the vast majority of rod-like, ionic LCs, all of the salts (except where *m* = 3 which do not show LC behaviour) form a SmA phase, with an additional SmB phase seen where X = BF_4_. The predominance of SmA phases is readily understood simply by understanding that the electrostatic forces between anions and cations will tend to keep everything ‘in register’, lending itself to lamellar organisation. While this lamellar organisation is confirmed by SAXS, the observed layer spacings are appreciably greater that the length of an individual cation, increasing as 130%, 150%, 160% of the cation length for X = Tf_2_N, OTf, BF_4_, respectively.^80^ This is indicative of the formation of a bilayer structure arising from the preferential association/segregation of the different terminal chain types. Such organisation is discussed further in a later section below.

**Fig. 19:**
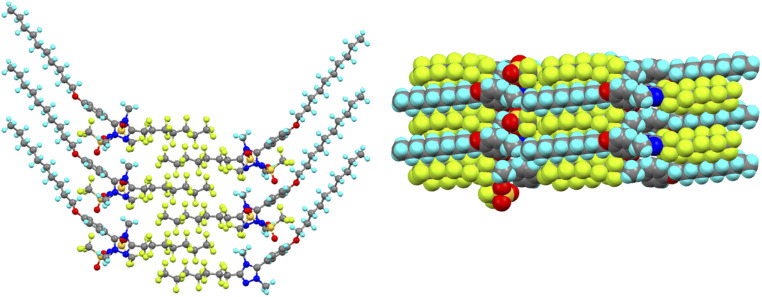
Packing in the solid state of: (a) [14-10,7][Tf_2_N] showing the additional organisation consequent on the presence of the fluorocarbon chains; (b) [14-10,9][OTf] where this additional organisation is absent and hydrocarbon and fluorocarbon chains are not segregated.

While understanding explicitly the danger of generalisations with respect to solid-state structures, nonetheless this level of organisation is reproduced in an extensive crystallographic study of the salts and an example is shown as Fig. 19a.^81^ However, there were also salutary cases where the two chain types did not de-mix in the solid state and an example of this is shown as Fig. 19b, showing the subtleties that can arise and cautioning against both sweeping generalisations and extrapolating solid-state organisation into fluid states.

**Fig. 20:**
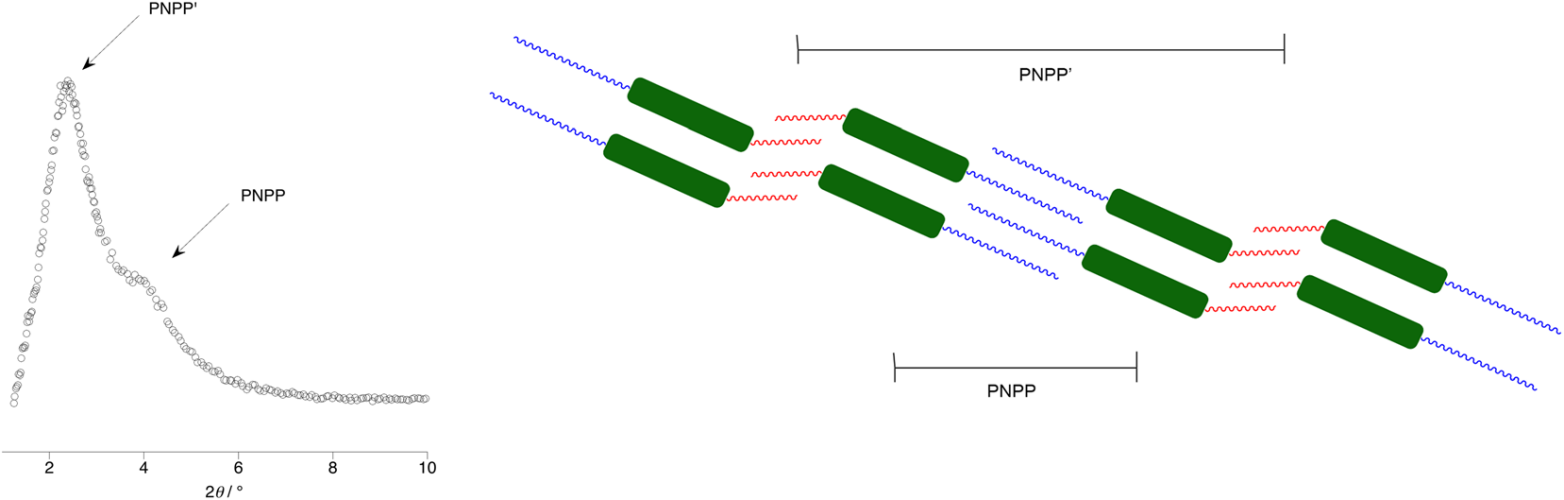
Schematic diagram to show: (a) the SAXS data for the [14-10,7][Tf_2_N] at 89 °C in the isotropic liquid phase showing the two independent reflections and (b) the two, independent spacings found in the isotropic phase of the triazolium salts with longer (C7 and C9) perfluoroalkyl chains (blue – hydrocarbon chain, red – fluorocarbon chain, green – triazolium cation). Anions, which will be associated closely with the cations, are omitted for clarity. Figure adapted from ref. 80.

However, perhaps the most remarkable observation and one that is rare if not unique in being well characterised, is that the triphilic nature of the organisation persists into the isotropic liquid. Thus, in ionic liquids (and indeed in the isotropic state above certain liquid crystal phases^82^), features associated with rather short-range ordering are seen using X-rays and neutrons in small-angle scattering experiments. The reflection seen at largest distances describes a very localised bilayer structure and, in the isotropic phase of each of the LC-forming salts (*i.e.* those with *m* = 7 or 9), this reflection had two distinct and independent components, which arise from two different correlation lengths. In the IL community, this reflection is known as the polar non-polar peak (PNPP) and the proposed organisation in these triazolium salts is shown in Fig. 20. The structural organisation that would commonly give rise to a PNPP is indicated as such, while the longer-spaced arrangement that is consequent on the hydrocarbon/fluorocarbon separation is noted as PNPP′.

### 4.4 More self-association driven by fluorocarbon chains

**Fig. 21:**
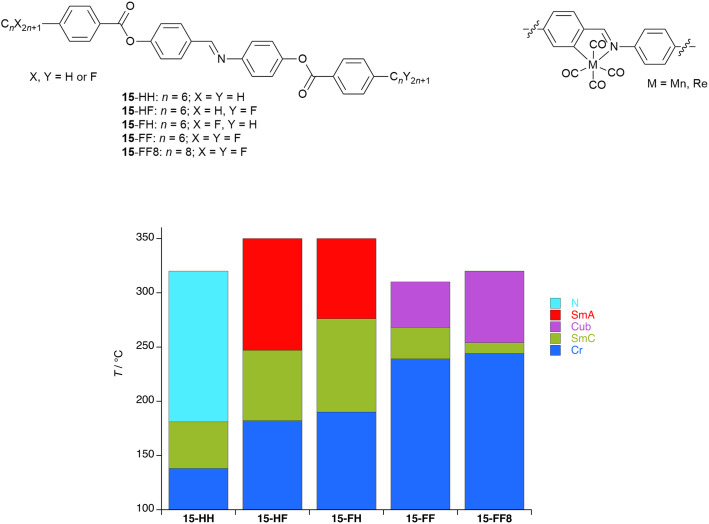
Structure of the four-ring imine ligands alongside an incomplete but indicative structure of the Mn^I^ and Re^I^ complexes that may be formed from them and a bar chart showing their liquid crystal phase behaviour.

Now taking further the idea of replacing hydrocarbon chains by their fluorocarbon equivalents, interesting consequences are observed from studies of extended rod-like systems with a central imine function, which were used for the preparation of metal complexes bearing a Mn^I^(CO)_4_ or Re^I^(CO)_4_ fragment (Fig. 21).^83–86^ As part of these studies, imine ligands were prepared with different combinations of hydrocarbon and fluorocarbon chains and the LC phase behaviour of some examples is plotted in Fig. 21.^87^

The starting point in the comparison is 15-HH, which melts to form a SmC phase after which a wide-range nematic phase exists to *ca.* 320 °C. If either of the terminal hydrocarbon chains is replaced by a fluorocarbon chain of the same length (15-HF or 15-FH), then the crystal phase is stabilised by around 40 °C, the SmC phase remains but the N phase is replaced by SmA with a clearing point some 30 °C higher. That the nematic phase is replaced by a SmA in these circumstances is unsurprising on the basis of the preceding discussion. However, when both hydrocarbon chains are replaced by fluorocarbons (15-FF), the melting point increases further, the SmC phase is narrowed in range and now the SmA phase is replaced by a cubic phase (Cub).

Most commonly found in the LC phases of surfactants, as the name suggests, cubic phases are three-dimensional LC phases with isotropic structures that can be described by different space-filling models.^88–91^ For many years they were extremely rare in non-surfactant LCs but, as the diversity of structures forming mesophases increased, so gradually they became more commonly seen. While in some types of LC materials their occurrence can be readily understood, exactly why they form in rod-like LCs is much less clear. However, we advanced a proposal that their formation required some degree of specific molecular self-association. These materials are consistent with that approach if it is recognised that the fluorocarbon chains would drive such behaviour.^92^

### 4.5 Exploiting amphiphilicity and chain volume in polycatenar liquid crystals

As described in the SI, polycatenar liquid crystals are rod-like mesogens with extended cores, functionalised with three or more terminal chains.^93^ The liquid crystal phase behaviour of five-chained (pentacatenar) and six-chained (hexacatenar) materials is dominated by the formation of columnar, and occasionally cubic, phases. However, while the phase behaviour of tricatenar materials is dominated by formation of the SmC phase, tetracatenar materials can show smectic, cubic and columnar phases.

**Fig. 22:**
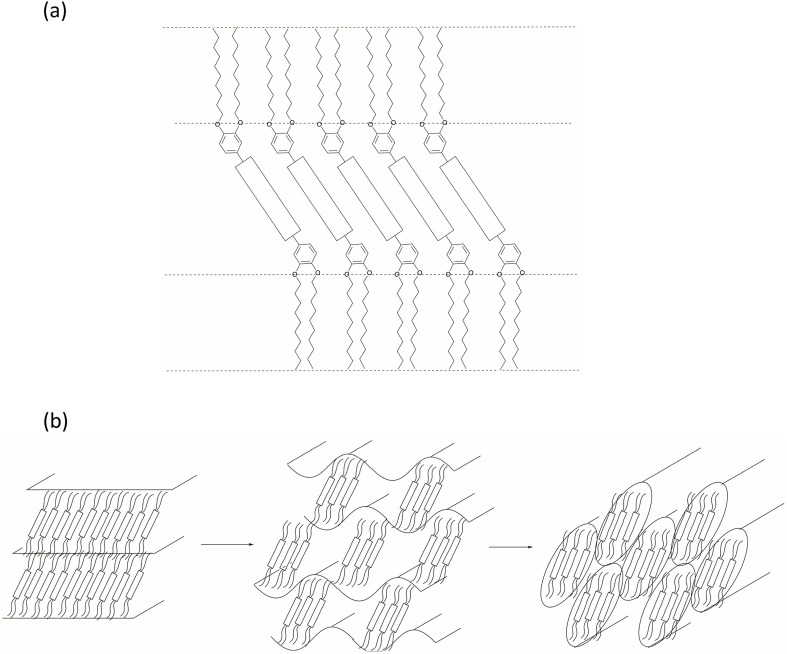
Figures to show: (a) the organisation in the SmC phase of tetracatenar mesogens and illustrates how the tilted core enables a planar core-chain interface; (b) the effect of increased terminal chain volume in a tetracatenar mesogen leading to undulating layers and finally columnar organisation. Fig. 22a is reproduced from ref. 92; Fig. 22b is adapted with permission from ref. 97. Copyright 2000 American Chemical Society.

Thus, different regioisomeric forms are possible for tetracatenar liquid crystals, but for the purposes of this discussion, only those substituted 3,4- on each terminal phenyl ring are considered. The LC phase behaviour of these compounds is fascinating and depends very strongly on terminal chain length (as a proxy for chain volume).^93^ As indicated in Fig. 22a, the compounds can form a SmC phase (sometimes with accompanying nematic phase) with the tilting of the extended core being driven by the desire for planarity at the core-chain interface. However, the effective volume of the chain can be increased either thermally through increased motion or by increasing its length, which at a critical chain length/volume disrupts the simple lamellar arrangement of the SmC and leads to a curved core-chain interface (Fig. 22b). Described in detail elsewhere,^92,94,95^ this leads either to the formation of a cubic phase or, more commonly, to formation of a columnar hexagonal (Col_h_) phase (see SI) and there are very strong analogies between this behaviour and that of amphiphilic surfactants as set out in ref. 96.

**Fig. 23:**
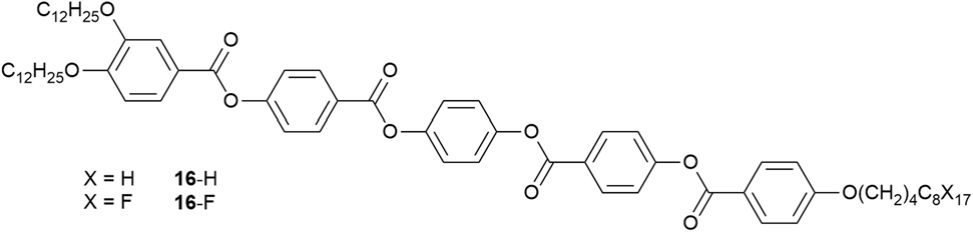
Structure of tricatenar LCs showing chain-dependent SmC phase structure.

A demonstration of self-association driven by a perfluorocarbon chain comes from work with tricatenar liquid crystals, which have an extended core, two terminal chains at one end of the molecule and one at the other. Here there are two dodecyloxy chains at one end of the core and a single C_12_ chain at the other (Fig. 23).^97^

**Fig. 24:**
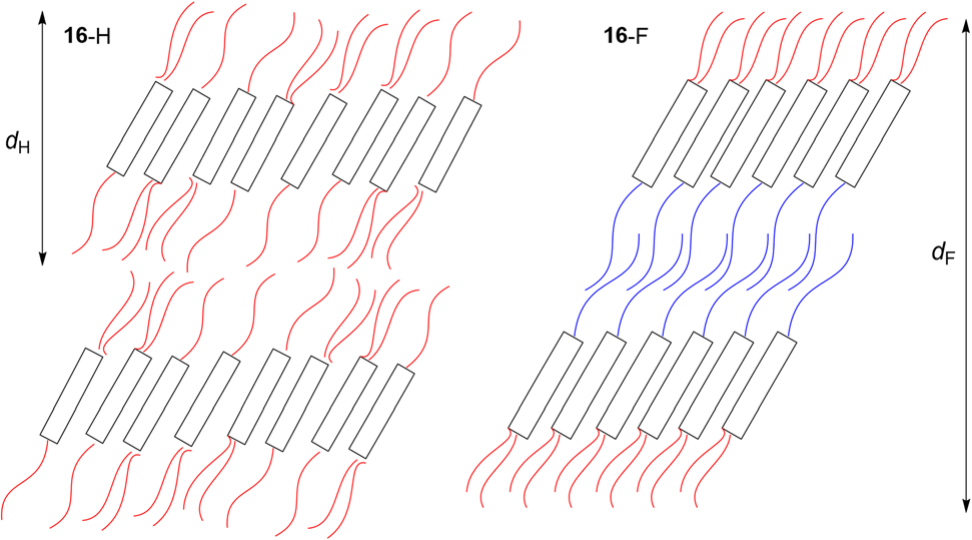
Schematic representation of the lamellar organisation in compounds 16-H and 16-F red ‘chains’ are hydrocarbon whereas blue ‘chains’ are fluorocarbon. The quantity *d*_H_ is the observed lamellar repeat for 16-H, while *d*_F_ is the observed lamellar repeat for 16-F.

The phase behaviour of such tricatenar LCs is dominated by the formation of the SmC phase and, as set out in the SI, the lamellar repeat distance measured by small-angle X-ray scattering will show the observed layer spacing to be less than the calculated molecular length (in this case *ca.* 63 Å) on account of the in-layer tilt (although calculations of the tilt angle need to be undertaken with care in the event that there is chain folding and/or interdigitation). Thus, the lamellar spacing determined for 16-H was 36.85 Å, while for 16-F it was 76.43 Å – slightly more than double. This can be understood by considering how the fluorous chain influences the lamellar organisation, which is illustrated in Fig. 24. Thus, while the organisation of 16-H is a simple monolayer, in 16-F there is a clear preference for self-association of the fluorocarbon chains, which may also be expressed as a clear preference for localised separation of hydrocarbon and fluorocarbon chains, causing the formation of a bilayer phase arising from triphilic organisation and hence a larger repeat distance.

However, in addition to self-association, another factor to be taken into account is that fluorocarbon chains occupy a greater volume than their hydrocarbon equivalents. Both effects can be seen by considering tetracatenar mesogens 17.^97^

**Fig. 25:**
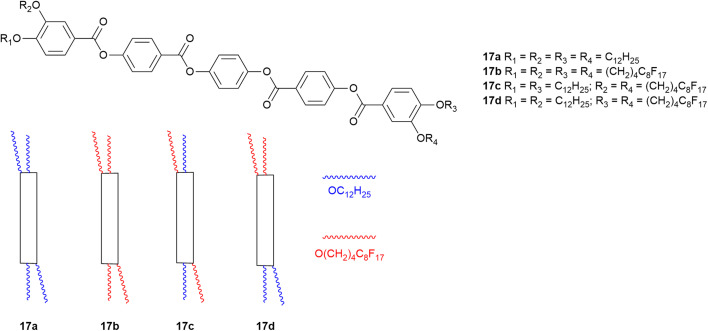
Structure of the tetracatenar liquid crystals under consideration including a schematic representation.

Thus, where R_1_=R_2_=R_3_=R_4_=C_12_H_25_ (17a, Fig. 25), the compound shows a SmC phase with a nematic phase above it. However, when all the terminal hydrocarbon chains are replaced with a semiperfluorinated chain of the same overall chain length (17b, Fig. 25), the increase in chain volume is sufficient to cause interfacial curvature and drive formation of a Col_h_ phase. Interestingly, if hydrocarbon and semiperfluorocarbon chains are forced together and unable to separate by virtue of being attached to the same ring (17c, Fig. 25), then the observed effect is only that of increased chain volume and a Col_h_ is observed. However, if the chains are deployed so as to generate an amphiphilic arrangement (17d, Fig. 25), then something quite different happens; the SmC phase is retained and the measured lamellar period is 75.9 Å, consistent with the segregated, bilayer organisation observed in 16-F (the two molecules are almost exactly the same length). However, the desire of the two chain types not to associate cannot lead to formation of a Col_h_ phase (in which it would be difficult to accommodate the different spatial and volume requirements). Instead, a lower-symmetry columnar rectangular phase (Col_r_) forms and its large lattice parameters reflect the optimum arrangement that will accommodate the competing factors (*a* = 160.3 Å, *b* = 92.5 Å).

### 4.6 Overcoming amphiphilicity

While the ability of fluorocarbon chains to direct organisation has been set out earlier, as observed by Gilmour *et al.* in their study of simple hydrocarbon/fluorocarbon mixtures,^74^ there is a critical point – in effect an upper consolute temperature – above which the two will mix. While formal consolute behaviour requires two components, the phase sequence of the gold complexes shown below shows something like this in a single-component system.

**Fig. 26:**
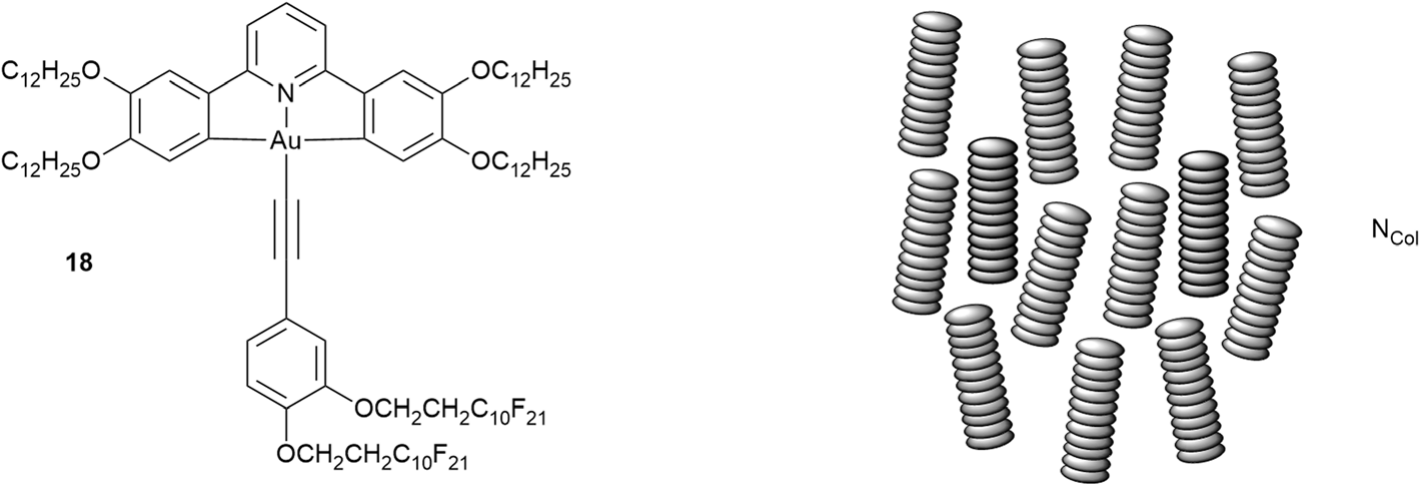
Structure and LC phase behaviour of an amphiphilic gold complex and the organisation in the N_Col_ phase.

The complex in question (18, Fig. 26), which is amphiphilic, arose from a study of luminescent LC complexes of gold(iii)^24,98^ and stood out on account of its remarkable phase behaviour:^99^Cr·88.4·Col_r_^1^·93.5·Col_r_^2^·139.4·N·156.1·Col_h_·201.3·Iso

**Fig. 27:**
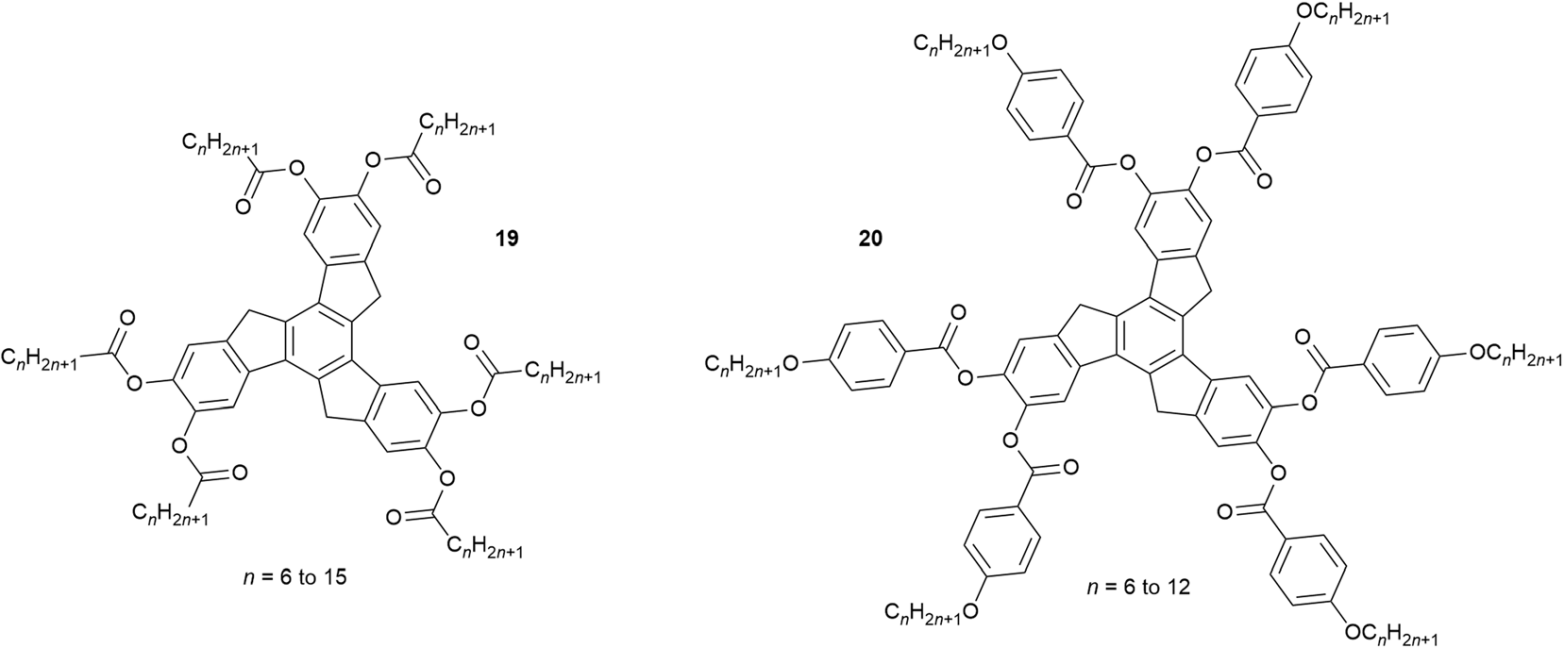
Disc-like truxene LCs showing an out-of-sequence nematic phase.

In the vast majority of cases, cooling a liquid crystalline material that has more than one mesophase leads (intuitively) to successively more ordered phases. However, the phase sequence for 18 shows that below the Col_h_ phase, rather than a more ordered columnar phase, there is in fact a (disordered) nematic phase and only at even lower temperatures does a (more ordered) Col_r_ phase form. As such, the nematic phase is very much ‘out of sequence’. Such a phenomenon is so rare in disc-like LCs, that the only previous reports relate to a small series of related truxenes (19, 20Fig. 27) reported in the early 1980s,^100–103^ which can be seen to bear no resemblance to the gold complex 18.

The behaviour is perhaps most easily understood by considering the cooling process. Using the idea of the consolute temperature, then above *ca.* 200 °C when the Col_h_ forms, the hydrocarbon and fluorocarbon chains evidently mix freely with no preference for association and so a high-symmetry Col_h_ phase forms. However, as the temperature falls then the chains will begin to exhibit their preference for self-association (localised de-mixing), leading to an amphiphilic organisation in the columns which cannot support hexagonal phase organisation. This situation is analogous to that of the tetracatenar LC 17d (above) which is also unable to sustain hexagonal phase organisation. However, the loss of hexagonal organisation does not result immediately in the formation of a lower-symmetry columnar phase. Instead, the assembled columns ‘melt’ from their hexagonal lattice positions, retain their orientational order and so form a nematic phase – the so-called N_Col_ phase, for which there is ample literature precedent.||||Note that a N_Col_ phase is much more likely than a ‘simple’ nematic phase of isolated, molecular discs, whose formation from the Col_h_ phase would require both the melting of the columns away from their lattice position and the melting of the columns themselves, with both events being reversed on formation of the Col_r_ phase.^104^ Note also that this mesophase is stable thermodynamically in the phase sequence and is not a metastable, transient phase. Then, as the temperature decreases further, amphiphilic self-organisation within the columns becomes established and the columns can re-establish a 2D lattice, now of (reduced) rectangular symmetry to allow for the additional organisation.

## 5. Concluding remarks

Fluorine is indeed a remarkable element and it is hoped that the examples chosen act as an accessible illustration of the effects that it can drive and the different ways in which it can exert itself. While many of the examples have been drawn from our own studies, there are innumerable groups that have worked on the specific deployment of fluorine in small-molecule LCs. It is not possible (neither was it intended) to do specific justice to their work, but when considering the structure-directing use of fluorocarbon chains, inspiration is inevitably drawn from the work Tschierske and co-workers^105–108^ and also in the way that fluorous chains were deployed in tapered and dendritic systems by Percec and his group.^109–113^ Nonetheless, it is hoped that the examples given from very systematic studies exemplify this general field of endeavour.

Of course, fluorocarbons are very much in the news at the present time, with so-called ‘forever chemicals’ turning up across the environment owing to their indiscriminate deployment over many years. We understand what these materials are^114^ and attempts at re-classification^115^ do not seem the way to accept the damage they have caused. However, work by Yang *et al.*^116^ has recently shown how these materials may be ‘digested’ and there are also recent media reports outlining some commercial approaches to dealing with perfluorocarbons in waste,^117^ so that there is some hope for remediation.

Indeed, both as F in a C–F bond and as CF_3_, fluorine has a role in, for example, a wide range of bio-active materials^118^ and, furthermore and as hinted in some of the work described above, it may have a role to play in cleaner catalysis in SILP systems. With the benefit of hindsight, it is possible to undertake such work responsibly and in a contained fashion, so that the potential benefits of fluorine can be realised without negative environmental consequences.^119^ Indeed, more broadly, this a challenge for our subject now and into the future.

## Author contributions

The article was conceived and written by D. B.

## Conflicts of interest

The authors declares no conflicts of interest.

## Supplementary Material

SC-OLF-D5SC05945C-s001

## Data Availability

No primary research results, software or code have been included and no new data were generated or analysed as part of this review. Supplementary information: a brief, high-level introduction to liquid crystals to cover the material discussed in the perspective. See DOI: https://doi.org/10.1039/d5sc05945c.
