# Supplementary material for: Employing fluorine for supramolecular control in self-assembled and self-organised molecular systems
Source: Chem Sci. 2025 Nov 4;16(47):22213–30. doi: 10.1039/d5sc05945c (PMC12612855; doi:10.1039/d5sc05945c)
Supplement: SC-016-D5SC05945C-s001 [file SC-016-D5SC05945C-s001.pdf]

Supplementary Information (SI) for Chemical Science.
This journal is © The Royal Society of Chemistry 2025

Employing Fluorine for Supramolecular Control in Self-Assembled and Self-Organised Molecular Systems

Duncan W. Bruce

Supporting Information

Introductory Material on Liquid Crystals

Background Material – Liquid Crystals

It is intended that this Perspective is a stand-alone article and that it be accessible to the widest readership. As such, it is appropriate to provide a short overview of some basic LC science in order to enhance appreciation of the material and is accompanied by a bibliography for further reading.

The liquid crystal (LC) phase is a true state of matter, existing between the solid phase and the liquid phase. It can be generated in one of two ways. For some materials (described below), mesophases are accessed by heating/cooling the compound – these are *thermotropic liquid crystals*. There are also systems where it is the addition of solvent, often in conjunction with temperature, that leads to the formation of the liquid crystal phase. These are termed *lyotropic liquid crystals* and are not the subject of this Perspective. Discovered at the end of the 19th century, it really took off in the 1970s following the commercialisation of low-cost, flat-panel liquid crystal displays that used mixtures of the cyanobiphenyl and cyanoterphenyl compounds prepared at the University of Hull by a team led by the late George Gray. Optimisation of mixtures for displays was carried out at the Royal Signals and Radar Establishment (RSRE) of the UK Ministry of Defence in Malvern, while manufacture was scaled up by BDH Chemicals in Poole.

Like the solid state that it borders, molecules in LC mesophases have some combination of orientational and translational order, while like the liquid state, LC mesophases have fluidity. The combination of order and fluidity means that the physical properties of mesophases are anisotropic and it is this that is exploited in their many applications.

LC mesophases are formed of molecules that are structurally anisotropic and are stabilised by the weak anisotropic dispersion forces that arise as a consequence of the molecular shape. There are three principle motifs that can give rise to LC phases – rods, discs and a bent shape (Figure S1).

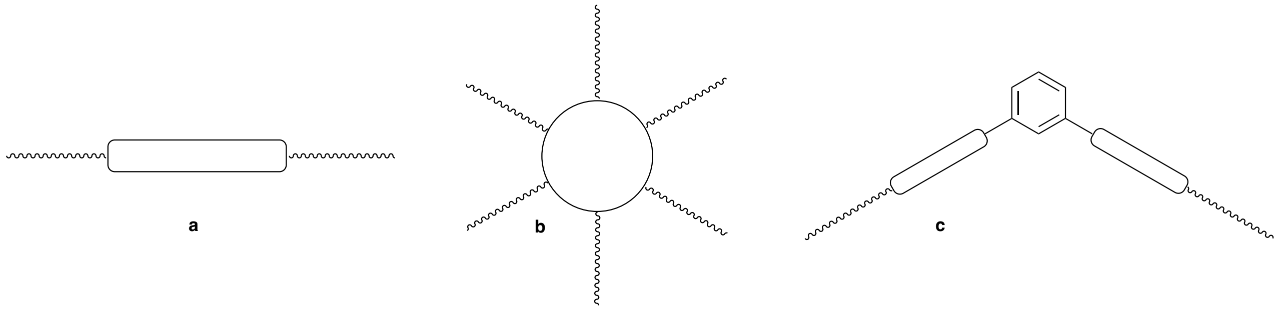

Figure S1 Three principle molecular motifs forming thermotropic liquid crystal phases: (a) rod, (b) disc, (c) bent-core.

Rod-like (or calamitic) liquid crystals (mesogens) have a unique, long axis and two shorter axes (Figure S2), the shape normally arising from the linking together of at least two, or more typically three, six-membered rings linked together either by σ-bonds or by some other linking group (X) that maintains the overall structural anisotropy (*e.g.* imine, vinyl, ester). The rings are most commonly aromatic (and may be heterocyclic), although unsaturated rings are not uncommon. One, and more commonly both, ends (A, B) of the molecule bear an alkyl chain that acts both to enhance the anisotropy and also, on account of its flexibility, to moderate the melting point.

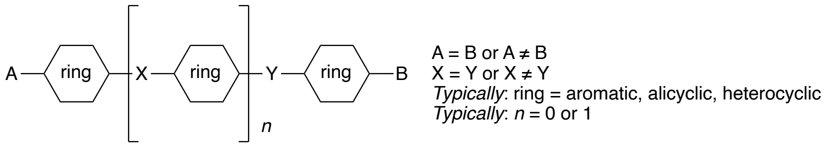

Figure S2 Indicative, idealised structure of a calamitic liquid crystals. Abbreviations discussed in the text.

Mesophases of Rod-like Mesogens

In forming LC mesophases, compounds *melt* from the solid state (Cr) into the LC phase and *clear* from the highest-temperature LC phase observed into the normal (isotropic – Iso) liquid state. Melting is also used to describe the transition from the solid state to the isotropic liquid and from one LC mesophase into another.

The most disordered phase (Figure S3) is the nematic phase (N) in which the long molecular axes are correlated in one direction (along the director, **n**), but where there is no translational ordering

of the molecules. To lower temperature, there are a series of smectic phases and, while there are very many variants, this account will describe only the most common, which are referred to in the Perspective. The smectic phases are abbreviated as Sm and this is spelled out in this paragraph; the literature shows that *e.g.* SmA and smectic A are used interchangeably. Thus, in the smectic A (SmA) phase, in addition to the orientational correlation, there is a degree of translational ordering that defines a layered structure. These layers are not normally well-defined and there is a high probability that individual molecules will diffuse between these layers. The director in the smectic A (SmA) phase is orthogonal to the layer direction. The smectic C (SmC) phase is similar to the smectic A (SmA) phase except that the molecules are tilted within the layers, generating a tilt angle (θ) defined as shown in Figure S3).

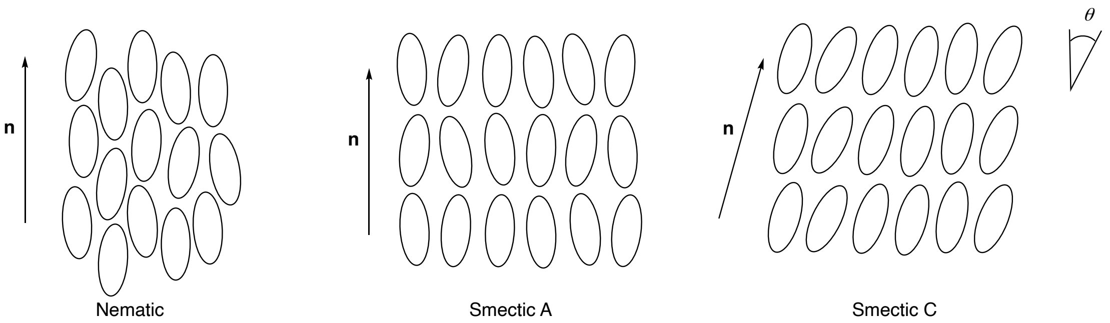

Figure S3 Schematic showing the nematic, SmA and SmC phase of calamitic liquid crystals indicating in each case the director **n**.

The nematic and tilted smectic phases have chiral analogues whose physical properties are influenced by the presence of chirality (introduced either through inclusion of a chiral moiety in the mesogen or by use of a chiral dopant which need not itself be LC in nature). Chirality is indicated by the addition of a '*' and should be used wherever the mesogens are chiral, irrespective of whether the phase itself is chiral (*e.g.* properties of the SmA phase are unchanged by the presence of chirality, although formally the symmetry of the phase is reduced). Chirality induces additional helical ordering and in the N* phase (known properly as 'chiral nematic' rather than cholesteric), the helix propagates perpendicular to the director. If the pitch of the helix is *p*, then a chiral nematic phase will reflect light of wavelength λ = *p*<*n*> where <*n*> is the refractive index of the material. This is the basis of liquid crystal thermometry as *p* increases with

temperature and *p*<*n*> tends to be on the order of visible light. The SmC* phase has lower symmetry (C_2) than its achiral analogue (C_{2h}) resulting in net spontaneous polarisation (P_s) along the layer direction. In the SmC* phase, the helix propagates perpendicular to the layer plane, but using surface treatment in thin (μm) cells can unwind the helix giving rise to a switchable structure with net ferroelectric polarisation.

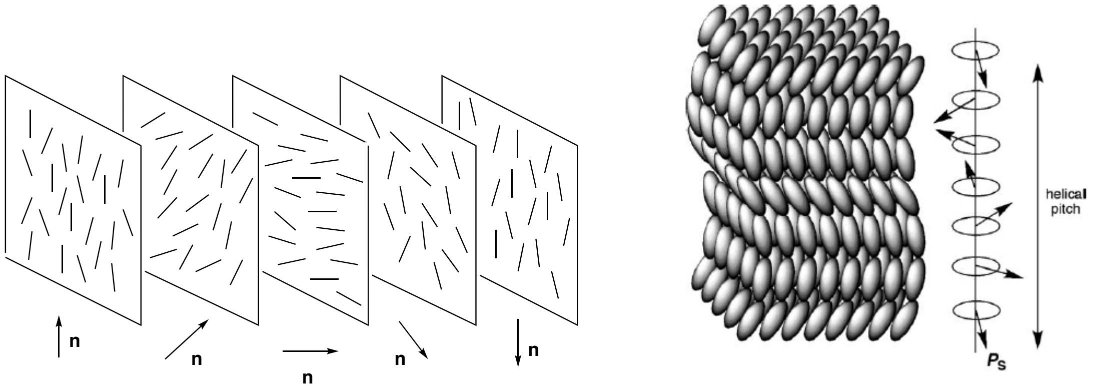

Figure S4 Schematic diagram of the N* phase (left) and the SmC* phase showing the helical precession of the spontaneous polarisation (right). SmC* figure reproduced from R. P. Lemieux, *Chem. Soc. Rev.*, 2007, **36**, 2033-2045 by permission of the Royal Society of Chemistry.

Mesophases of Disc-like Mesogens

Disc-like mesogens can show one of two types of nematic phase (Figure S5), neither of which is particularly common. These are the so-called 'discotic nematic' – N_D – and the columnar nematic, N_{Col}. The N_D phase is a direct analogues of the nematic phase of calamitic mesogens inasmuch as it is the unique (short) axis that is correlated orientationally about the director, while in the N_{Col} phase, the 'unit' that correlates orientationally is a stack of discs.

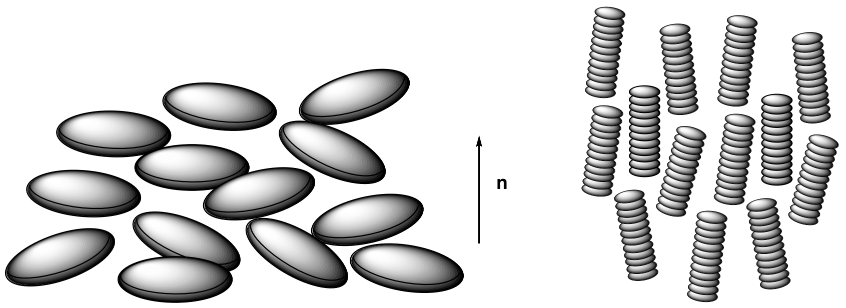

Figure S5 Left: schematic of the N_D phase; right: N_{Col} phase.

In addition, there is a large family of columnar phases (Figure S6). These phases are constituted of stacks of discs which are in turn disposed on a 2D net of a particular symmetry. The most common examples are columnar hexagonal (Col_h), columnar rectangular (Col_r) and columnar oblique (Col_o). Note that there are different 2D symmetries that can be classified as Col_r. The precise phase symmetry is often not determined (insufficient reflections from small-angle X-ray scattering), but where it is, then it can be added to the labelling (*e.g.* Col_r(*p*2*gg*)).

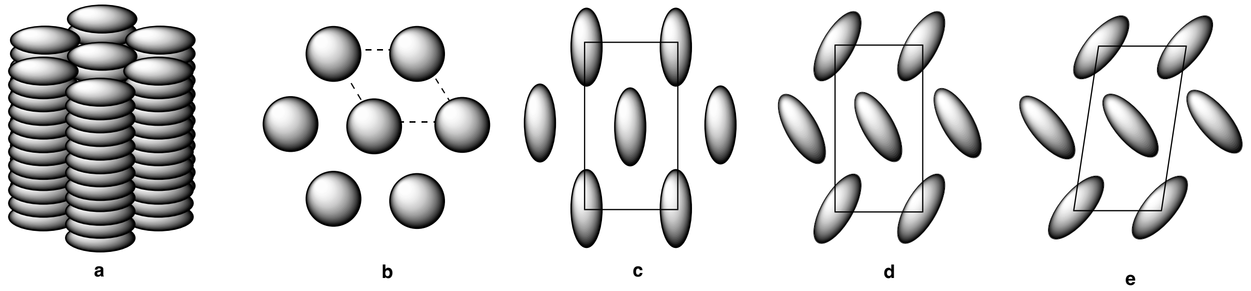

Figure S6 (a) Side-on view of the Col_h phase; top-down view of (b) Col_h phase; (c) Col_r(*c*2*mm*) phase; (d) Col_r(*p*2*gg*) phase; (e) Col_o phase.

Mesophases of Polycatenar Mesogens

As described in the Perspective, polycatenar liquid crystals consist of a long, rigid, rod-like core with between three (*tri*catenar) and six (*hexa*catenar) terminal chains. Depending on the number, length and relative disposition of the chains, they can form nematic, smectic (normally SmC), cubic and columnar phases. The columnar phases are in one way exactly the same as those shown in Figure S6 if the columnar phase is understood to be described by its overall structure and symmetry. However, the different is the way in which the constituent molecules are organised within the columns. For columnar phases of disc-like materials, the representation in Figure S6a is perfectly good – discs simply stacked into columns. However, for polycatenar materials the picture is a little less straightforward and it is both unhelpful, and in truth somewhat inaccurate, to consider 'slices' of column that may contain some defined number of molecules. Indeed, this

author would dissuade the use of such an approach. Those wishing a more detailed understanding are referred to: B. Donnio *et al., J. Am. Chem. Soc.*, 2004, **126**, 15258-15268.

Characterisation of Liquid Crystal Mesophases

Three techniques are used commonly: polarised optical microscopy, differential scanning calorimetry (DSC) and small-angle X-ray scattering (SAXS). A very wide range of other techniques exist that can probe particular physico-chemical responses. SAXS is the only technique referred to in the Perspective and is introduced briefly here.

In order to carry out SAXS in the LC phase, the sample is normally held in a capillary in a furnace whose temperature can be controlled. X-Rays pass through the sample, are diffracted and the diffracted radiation is recorded using a detector, which may be able to collect in 1D or 2D; in most cases 1D data are used in interpretation (examples in Figure S7). As the LC phase is fluid, the information content is normally rather low, reflections are generally few and not sharp, and so the data need to be used in conjunction both with other characterisation techniques (polarised optical microscopy, DSC), wider LC knowledge and, above all, common sense. It is pointed out here that the correct deployment of SAXS as a technique and the interpretation of the data obtained is not trivial. Furthermore, data must be collected at the temperature at which the mesophase of interests exists and cooling to room temperature is the hope and expectation that the phase may persist is to be avoided.

At small values of 2θ[*] (*e.g.* $0.5 < 2\theta < 3$ equivalent to a spacing between *ca* 88 and 29 Å), reflections for smectic phases (00*l*) will reflect the observed layer periodicity (*d*), which for a SmA phase is normally quite close to the molecular length. In the tilted SmC phase, the observed later

[*] Reflections are given here as 2θ as this has, for many years, been the more common unit employed in the LC literature and is related to the spacings *d* through the Bragg equation. However, 2θ is wavelength dependent and so increasingly the wave vector *q* (defined as $2\pi/d$) is employed. Like 2θ, *q* reflects reciprocal space (units of $Å^{-1}$) and so small values of *q* equate to large values of *d*.

spacing will always be less than the molecular length (*l*) and, if it assumed that there are no other effect to be considered, then the tilt angle may be calculated as $\cos^{-1}(d / l)$. Of course, there are other effects that can be at play, so that bilayer formation (see Perspective) is one possibility and chains can also be interdigitated and/or folded, which also reduces the apparent molecular length. Note that in most cases, higher-order reflections – (002), (003) *etc.* – are not observed in fluid smectic phases ,although this is not always true in materials where there are fluorocarbon terminal chains and where the smectic phase is of an ionic material.

In the same low-angle region, reflections (*hk*) can be observed for the 2D plane groups that characterise columnar phases. As an example, for hexagonal phases (plane group *p*6*mm*), then reflections follow the rule in Eqn. 1:

$$d_{obs} = 2\sqrt{(h^2 + k^2 + hk)}$$

so that observed spacings are in the ratio 1 : √3 : √4 : √7 corresponding to the (10), (11), (20) and (21) reflections. The presence of *both* the (10) *and* the (11) reflection is the minimum required to identify a mesophase as hexagonal and in many cases no more than two or three reflections are observed.

Larger values of 2θ represent nearest neighbour spacing and where mesogens bear alkyl chains, then a very broad reflection is observed centred around $2\theta \approx 20°$ (corresponding to *ca* 4.4 Å). However, fluorocarbon chains are more voluminous and, where they dominate the nearest-neighbour spacing, then this wide-angle reflection is observed at smaller angles ($2\theta \approx$16-17° corresponding to *ca* 5.4 Å).

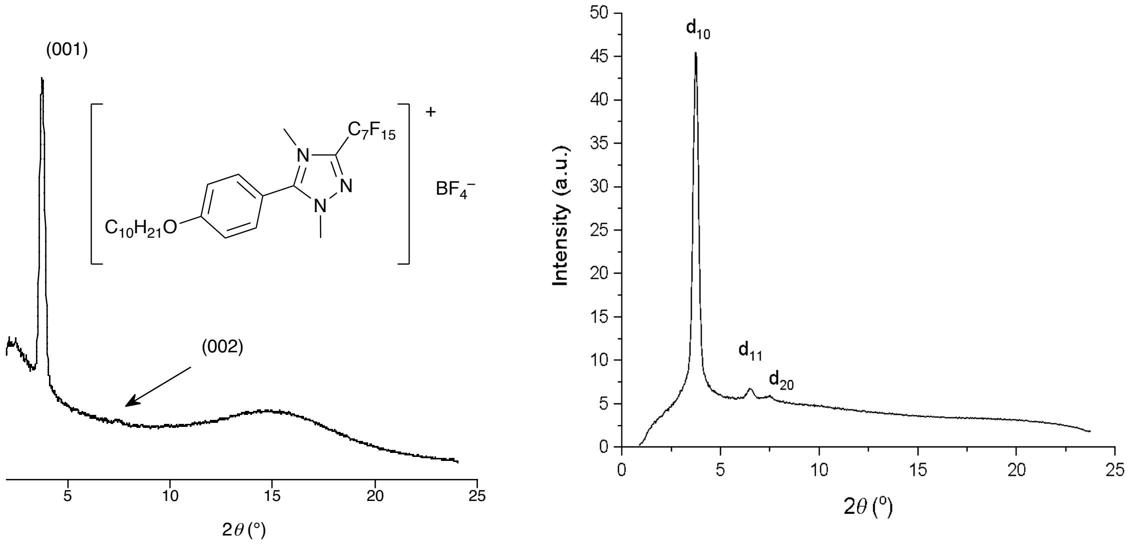

Figure S7 Small-angle X-ray reflections from: (a) the SmA phase of the illustrated salt, showing the lamellar (001) and (002) reflections and nearest-neighbour separation at *ca* $2\theta = 15°$ (see text – from A. Riccobono *at al.*, *J. Mol. Liq.*, 2021, **321**, 114758 – replotted from the original data); (b) the Col_h phase of a gold(III) mesogen from R. R. Parker *et al.*, *J. Mater. Chem. C*, 2021, **9**, 1287).

It is noted that SAXS is generally not helpful in the study of the nematic phase, for while a very broad reflection can often be observed, there is no positional periodicity.

Anisotropy of Physical Properties

One of the things that makes liquid crystals so interesting and which is fundamental in their applications, is the anisotropy in their physical properties. Consider a nematic phase of calamitic LCs as shown in Figure S8, where *Q* represents some physical property that, as shown in the diagram, is directionally dependent. For example, consider the simple cyanobiphenyl LC shown in the figure. It is evident that this molecule has a dipole moment that is coincident with its long molecular axis. In a nematic phase, such molecules are organised in which a way that molecular dipoles are arranged on average antiferroelectrically so that there is no net polarisation. Thus, in a nematic phase composed of which molecules, it is evident that if the quantity in question is the dielectric susceptibility, ε, then $\varepsilon_{\|} > \varepsilon_{\perp}$ so that $\Delta\varepsilon > 0$, *i.e.* the phase has a positive dielectric anisotropy. However, if the difluoroterphenyl in the figure is considered, then it is straightforward

to see that, with a dipole moment perpendicular to the long axis and organised in a nematic phase, then $\varepsilon_{\perp} > \varepsilon_{||}$ so that $\Delta\varepsilon < 0$, *i.e.* the phase has a negative dielectric anisotropy.

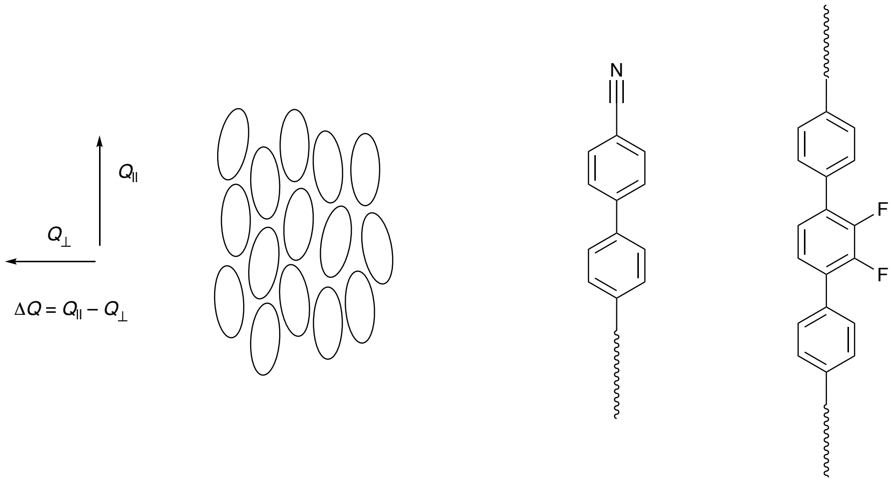

Figure S8 Left: schematic diagram to show the origin of the anisotropy of physical properties in a nematic phase; right: two examples of LCs as discussed in the text.

How might this be applied? Consider Figure S9, which shows, on the left, a schematic of a nematic phase confined in a cell of some sorts and with the director arranged perpendicular to the cell plates. In the example, it is assumed that $\Delta\varepsilon > 0$, so that on application of an external field (right-hand side), the molecules couple with that field and their orientation changes. Such a switching effect is the basis of reorientation of LC molecules within a phase that gives rise to the optical contrast used in commercial, flat-panel displays.

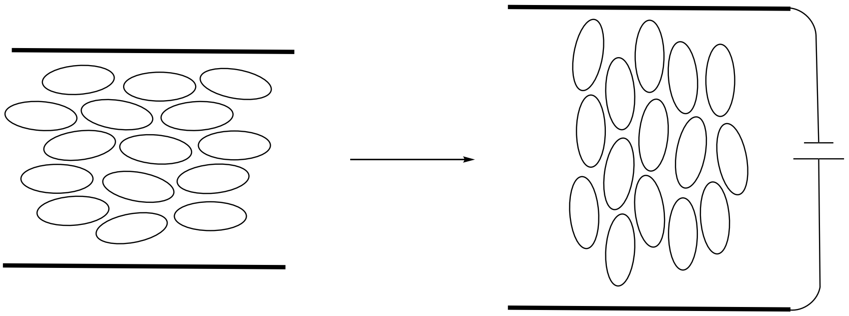

Figure S9 Schematic to show to change in orientation on molecules in a nematic LC mesophase on application of a potential across a cell for a material with a positive dielectric anisotropy. Note that: (i) while the applied field is shown for simplicity as DC, in reality AC fields are used (recall that the phase is dipolar and not polar), (ii) the cartoon shows total alignment of all molecules, whereas in reality those molecules in the centre of the cell will show such alignment while those closer to the alignment layer on the cell surface will retain some of that surface alignment.

Other common properties whose anisotropy is of interest include diamagnetism (χ) and polarisability (α), the latter of which in turn relates to refractive index (n) where the anisotropy (Δn) is termed the birefringence. Note that the anisotropy is a bulk property of the mesophase, even if it depends also on a molecular property and so this illustrates well the Simon definition of a molecular material mentioned in the Introduction in the Perspective.

Bibliography

Handbook of Liquid Crystals 2nd Edition, Eds. J. W. Goodby *et al.*, VCH, Weinheim, 2014 (eight-volume set).

Textures of Liquid Crystals, I. Dierking, VCH, Weinheim, 2003.

Smectic Liquid Crystals – Textures and Structures, G. W. Gray and J. W. G. Goodby, Leonard Hill, Glasgow, 1984. Book is long out of print and, while it does not cover so many new phases and phase types that are now known, it is invaluable for what it *does* contain.
